# Biomarker selection for medical diagnosis using the partial area under the ROC curve

**DOI:** 10.1186/1756-0500-7-25

**Published:** 2014-01-10

**Authors:** Man-Jen Hsu, Yuan-Chin Ivan Chang, Huey-Miin Hsueh

**Affiliations:** 1 Institute of Statistical Science, Academia Sinica, Taipei 11529, Taiwan; 2 Department of Statistics, National ChengChi University, Taipei 11605, Taiwan

**Keywords:** Discriminatory power, Hypothesis testing, Optimal linear combination, Partial area under ROC curve, Stepwise biomarker selection

## Abstract

**Background:**

A biomarker is usually used as a diagnostic or assessment tool in medical research. Finding an ideal biomarker is not easy and combining multiple biomarkers provides a promising alternative. Moreover, some biomarkers based on the optimal linear combination do not have enough discriminatory power. As a result, the aim of this study was to find the significant biomarkers based on the optimal linear combination maximizing the pAUC for assessment of the biomarkers.

**Methods:**

Under the binormality assumption we obtain the optimal linear combination of biomarkers maximizing the partial area under the receiver operating characteristic curve (pAUC). Related statistical tests are developed for assessment of a biomarker set and of an individual biomarker. Stepwise biomarker selections are introduced to identify those biomarkers of statistical significance.

**Results:**

The results of simulation study and three real examples, Duchenne Muscular Dystrophy disease, heart disease, and breast tissue example are used to show that our methods are most suitable biomarker selection for the data sets of a moderate number of biomarkers.

**Conclusions:**

Our proposed biomarker selection approaches can be used to find the significant biomarkers based on hypothesis testing.

## Background

A biomarker is a biological indicator showing the absence, presence, or the condition of a disease, and it can be used to determine the status of a subject, the effectiveness of a treatment, and so on. Ideally, a biomarker with both high sensitivity and specificity for accurate prediction is preferred. However, it is not easy to find such a biomarker in practice. Combining biomarkers provides an alternative to improve the performance of those individual biomarkers that are currently available. The serum prostate-specific antigen PSA is a typical example. It is a well-accepted prognostic biomarker used to screen for prostate cancer. However, this test has a low specificity and therefore might lead to over-diagnosis and over-treatment. In addition to PSA, several other alternatives have also been investigated [1]. Nevertheless, there is no single alternative which outperforms PSA, and therefore most investigators propose the use of a combination of PSA and other biomarkers. The combination of PSA and percent-free PSA is an alternative method [2]. Recently, due to significant advances in biotechnology, many genetic and genomic biomarkers have been discovered that could be potential candidates [3]. Once their clinical evidence is validated, integrating multiple biomarkers in order to obtain a better prediction will become an essential and important task.

The ROC curve is the most popular graphical tool for evaluating the diagnostic power of a biomarker. It provides an exhaustive look at the trend of sensitivity over all cutoffs, and thus provides information about the relationship between the sensitivity and the specificity of a biomarker. However, the abundance of information it provides makes the comparison between biomarkers difficult, because the underlying ROC curves are often likely to cross. The area under the ROC curve (AUC), which integrates the curve over all cutoffs, is proposed for an efficient summarization. This criterion can be extended by giving different weights at various cutoffs according to, for example, the cost resulting from the prediction error in the diseased or in the non-diseased population, and the prevalence rate of the disease [4]. In some applications, investigators focus only on a part of the curve. For example, a high level of specificity is required for a biomarker serving as a population screening tool. As a consequence, a biomarker is assessed on the partial area under the ROC curve (pAUC) in a region of specificity above a certain level [5-7].

This study focuses on combining multiple continuous-scaled biomarkers into one single diagnostic or predictive rule for a disease with emphases on assessment of each biomarker. For better interpretability, we propose the use of a linear combination for summarization. The discriminatory power of a linear combination of biomarkers is evaluated based on the pAUC. The optimal linear combination, which provides the best discriminatory power among all combinations, is the target solution of research interest.

In the presence of multiple biomarkers, a traditional method of medical diagnosis is to fit a multiple logistic regression model to the data set. An example of this is the study of outcome prediction of aneurysmal subarachnoid hemorrhage (aSAH) patients [8]. Alternatively, seeking the maximal discriminatory power, the explicit form of the best linear combination in terms of AUC under a binormal model is derived [9]. Following their study, a solution that is superior to all others in certain scenarios when a high specificity or a high sensitivity is required was found [10]. Nevertheless, these scenarios are not universal. The use of empirical AUC estimates in finding the optimal linear combination was proposed [11,12]. In our earlier study, we found that not only the analytical derivation, but also the computation, became much more complicated with the use of the pAUC criterion [13].

When an optimal linear combination is available, the solution is useful in evaluating either the entire biomarker set or one specific biomarker in the set. For example, the maximal pAUC of a biomarker set provides the best discriminatory power that the biomarker set can achieve. If even the best linear combination does not have a significant discriminatory power, none of the biomarkers should be considered to be associated with the disease. In addition to the global predictability, some insights on the importance of an individual biomarker can be obtained from the coefficients in the optimal linear combination. If a coefficient is nearly zero, the corresponding biomarker contributes little to disease diagnosis and is regarded as less important. In this study, we propose three testing procedures based on the optimal linear combination maximizing the pAUC for assessment of the biomarkers.

The proposed statistical tests will be embedded in two stepwise biomarker selection methods to identify biomarkers of statistical significance. It’s known that a classification is parallel to a diagnostic rule. Recently, in order to deal with big data several algorithm-based classification approaches have been proposed which also directly use either AUC or pAUC as the objective function [14-21]. The computational feasibility and efficiency are usually the major considerations in development of the methods. One popular way is to add some penalty in the optimization to stabilize the calculation. The penalization naturally leads to variable selection, which is a desirable outcome in an analysis of a huge data set. In contrast, we consider the conventional stepwise selection methods, which select or discard a biomarker on the basis of the statistical significance. However, acquiring the evidence of significance necessitates intensive computation. Therefore, our methods are most suitable for the data sets of a moderate number of biomarkers.

The paper is organized as follows: In the first part of Section (Methods), the sample version of the optimal linear combination will be defined. The testing procedures for the global and individual discriminatory power will be proposed in the second part of Section (Methods). Furthermore, two biomarker selection approaches adopting the proposed tests will be developed in the third part of Selection (Methods). Numerical results, including an intensive simulation and real example analysis, are given in the first part and the second part of Section (Results). We then conclude this paper with a discussion in Section (Discussions). Finally, conclusions are given in Section (Conclusion).

## Methods

Let ***X*** be a random vector of *p* biomarkers related to the disease of a subject, and *D* be the binary disease status, where *D* = 1 indicates a subject from the diseased population, and *D* = 0 indicates a subject from the non-diseased population. Suppose

$$X\left|D=d∼\mathrm{MVN}\left(\mu_{d},\Sigma_{d}\right),d=0,1,\right)$$

where the covariance matrices **Σ**_**0**_ and **Σ**_**1**_ are positive definite. For any given real vector ***a*** ∈ *ℝ*^p^, the linear combination of *p* biomarkers, ***a***^T^ ***X***, has a distribution as follows:

$$a^{T}X\left|D=d∼N\left(a^{T}\mu_{d},Q_{d}\right)\right),$$

where *Q*_*d*_ *=* ***a***^***T***^**Σ**_***d***_ ***a***, for *d* = 0,1. Let Ф(·) denote the cumulative distribution function of *N*(0,1) and Ф^-1^(·) be its inverse function. Also *c*(*u*) = Φ^- 1^(1 - *u*) and **Δ**_*μ*_ = ***μ***_1_ - ***μ***_0_, then for a given threshold at specificity (1-*u*), the sensitivity of ***a***^*T*^ ***X*** is equal to

$$F\left(a,u\right)=\Phi \left(\frac{a^{T}\Delta_{\mu }-c\left(u\right)\sqrt{Q_{0}}}{\sqrt{Q_{1}}}\right).$$

Therefore, for a given specificity region (1-t,1) for some predetermined *t* ∈ (0,1), the partial area under the ROC curve (pAUC) of the linear combination, ***a***^T^ ***X***, is equal to

(1) $$\mathrm{pAUC}\left(a\right)=\int_{0}^{t}F\left(a,u\right)\;\mathrm{du}.$$

Similar to the AUC, the pAUC has the scale invariant property. For identification purposes, in this study the search for the optimal linear combination vector is restricted to the hyper-sphere with a unit radius. Let a^*^ be such a pAUC maximizer; that is,

$$a^{*}=\arg\;\max_{a\in E_{p}}\;\mathrm{pAUC}\left(a\right),$$

where *E*_*p*_ = {***a*** |‖ ***a*** ‖ = 1, ***a*** ∈ ℝ^*p*^}.

Assume two independent random samples are drawn from the non-diseased and diseased populations. Let *n*_0_ and *n*_1_ be the sample sizes of the non-diseased and diseased groups, respectively, and denote their minimum as *n* = min {*n*_0_,*n*_1_}. Under the normality assumption, the maximum likelihood estimates (MLEs) are employed in a sample version of the optimization problem, when the population parameters are unknown. The estimated mean vectors and covariance matrices are respectively denoted as follows: ${\hat{\mu }}_{0}$, ${\hat{\mu }}_{1}$, and ${\hat{\Sigma }}_{0}$, ${\hat{\Sigma }}_{1}$. Moreover, let ${\hat{\Delta }}_{\mu }={\hat{\mu }}_{1}‐{\hat{\mu }}_{0}$ and ${\hat{Q}}_{d}=a^{T}{\hat{\Sigma }}_{d}a$, for *d* = 0,1. Replacing the unknown parameters in Equation (1) by their corresponding MLEs, we have a sample version of the pAUC below:

(2) $$\begin{array}{l} {\hat{\mathrm{pAUC}}}_{n}\left(a\right)=\int_{0}^{t}{\hat{F}}_{n}\left(a,u\right)\mathrm{du},\\ \; \end{array}$$

where

$${\hat{F}}_{n}\left(a,u\right)=\Phi \left(\frac{a^{T}{\hat{\Delta }}_{\mu }-c\left(u\right)\sqrt{{\hat{Q}}_{0}}}{\sqrt{{\hat{Q}}_{1}}}\right).$$

Thus, the coefficients a^*^ are estimated by the maximizer of Equation (2):

$${\hat{a}}_{n}=\arg\;\max_{a\in E_{p}}\;{\hat{\mathrm{pAUC}}}_{n}\left(a\right).$$

The next theorem shows that the sample pAUC maximizer ${\hat{a}}_{n}$, is strong consistent.

**Theorem 1:** Suppose that the conditional distribution of  ***X*** |*D* = *d* follows *N* (***μ***_*d*_, **Σ**_*d*_) and **Σ**_*d*_ is positive definite for *d =* 0,1. Assume that *pAUC***(*a*)** in Equation (1) has a unique maximizer a^*^ in *E*_*p*_. Then the maximizer, ${\hat{a}}_{n}$, of the sample pAUC, ${\hat{\mathrm{pAUC}}}_{n}\left(a\right)$, in Equation (2) converges to a^*^ with probability 1 as *n* → *∞*. (The proof is given in Additional file 1).

Previously, we found that the pAUC function sometimes has local extrema or multiple maxima [13]. Therefore, we proposed a multiple-initial algorithm, which utilizes multiple initial points in a conventional optimization algorithm, to reduce the risk of not finding the global maximum. The uniqueness of the maximum is assumed in Theorem 1 to ease the complications brought on by the existence of multiple maxima.

In real applications, occasionally the calculated best linear combination had a low pAUC value, or some coefficients in the best linear combination were found to be nearly zero. Numerically, the relevant biomarkers might have a limited contribution to the disease prediction. In the following section, we will discuss how to assess the significance of biomarkers in terms of their discriminatory power. The proposed testing procedures will be utilized in our biomarker selection approaches to find a compact biomarker set which consists of only significant biomarkers for disease diagnosis.

## Hypothesis testing and biomarker selection

### Testing the discriminatory power

When an optimal linear combination is available, the solution is useful in evaluating either the entire biomarker set or one specific biomarker in the set. The first hypothesis testing problem of interest is to assess the overall discriminatory power of a biomarker set through its maximal pAUC, which is the best discriminatory power that the biomarker set can achieve. Once the overall diagnostic power is “statistically confirmed,” the next important issue is to evaluate the contribution of each biomarker. This type of information can provide more insight about the causal relationship between each biomarker and the disease. In this subsection, the statistical procedures for testing the discriminatory power of a set or of an individual biomarker are developed.

Considering only the class of linear combinations, we evaluate the global discriminatory power of a set of *p* ≥ 1 biomarkers, ***X***_,_ by testing the following hypotheses:

*H*_0*,g*_: *The biomarker set has no discriminatory power to the disease*

versus

*H*_1*,g*_: *The biomarker set has a discriminatory power to the disease.*

The null hypothesis *H*_0,*g*_ is true if the optimal linear combination of the biomarker set has no discriminatory power. Or equivalently, the maximal pAUC that the set can achieve through its linear combinations is not greater than the reference limit *t*^2^/2, which is the pAUC value of the non-informative diagnosis with a diagonal ROC curve. That is,

$$H_{0,g}:\mathrm{pAUC}\left(a^{*}\right)\leq \frac{t^{2}}{2}\mathrm{versus}\;H_{1,g}:\mathrm{pAUC}\left(a^{*}\right)>\frac{t^{2}}{2}.$$

By maximizing the sample pAUC defined in Equation (2), we obtain the maximal sample pAUC and use it as the test statistic. That is,

$$\begin{array}{ll} T_{g} & =\max_{a\in E_{p}}{\hat{\mathrm{pAUC}}}_{n}\left(a\right)={\hat{\mathrm{pAUC}}}_{n}\left({\hat{a}}_{n}\right)\\ & =\int_{0}^{t}\Phi \left(\frac{{\hat{a}}_{n}^{T}{\hat{\Delta }}_{\mu }-c\left(u\right)\sqrt{{\hat{Q}}_{0}}}{\sqrt{{\hat{Q}}_{1}}}\right)\mathrm{du}. \end{array}$$

In fact, *T*_*g*_ is the estimated pAUC of the best linear combination ${{\hat{a}}_{n}}^{T}X$. The null hypothesis *H*_0,*g*_ is rejected if *T*_*g*_ is sufficiently large.

Due to the complex formulation of the test statistic, the null distribution and the right-tailed critical value are estimated by a parametric bootstrapping method. Under *H*_0,*g*_, ***X*** has a common multivariate-normal distribution in the two population groups. The common mean and covariance matrix are estimated from the pooled sample, and are denoted as ${\tilde{\mu }}_{p},{\tilde{\Sigma }}_{p}$. Consider drawing two independent random samples of size *n*_1_ and *n*_0_ from the estimated common null distribution, $\mathrm{MVN}\left({\tilde{\mu }}_{p},{\tilde{\Sigma }}_{p}\right)$. Then use the bootstrap samples to find the test statistic, say ${T_{g}}^{\left(b\right)}.$ Repeat the sampling *B* times. The critical value at the significance level α is then equal to the 100 (1-α)^th^ percentile among these ${T_{g}}^{\left(b\right)}$ values. The null hypothesis *H*_0_,_*g*_ is rejected if *T*_*g*_ is greater than or equal to the critical value.

When a set consists of only one biomarker, say *X*_*i*_*,* the global effect becomes the marginal discriminatory power of *X*_*i*_ alone. Using the correspondent pAUC to describe its discriminatory power, we can assess the biomarker by testing the following hypothesis:

$$H_{0,m}:\mathrm{pAUC}\left(1_{i}\right)\leq \frac{t^{2}}{2},$$

where **1**_*i*_ is the vector having zero components, except for a 1 in the position correspondent to *X*_*i*_. Again, we use the estimated pAUC value as the test statistic,

$$\begin{array}{ll} T_{m,i} & ={\hat{\mathrm{pAUC}}}_{n}\left(1_{i}\right)\\ & =\int_{0}^{t}\Phi \left(\frac{\left({\hat{\mu }}_{1,i}-{\hat{\mu }}_{0,i}\right)-c\left(u\right)\sqrt{{\hat{\sigma }}_{0,i}}}{\sqrt{{\hat{\sigma }}_{1,i}}}\right)\mathrm{du}, \end{array}$$

where ${\hat{\mu }}_{1,i},{\hat{\Sigma }}_{1,i}$ and ${\hat{\mu }}_{0,i},{\hat{\Sigma }}_{0,i}$ are the MLEs of the mean and variance of *X*_*i*_ in the two groups. The critical value is determined by the parametric bootstrapping method described previously. Here, only one single biomarker is involved, so the computation is even simpler.

When multiple biomarkers, ***X*** are simultaneously taken into account, we consider assessing one specific biomarker given the existence of other biomarkers. Let $X^{T}=\left(X_{i-}^{T},X_{i}\right)$, where *X*_*i*_ denotes the target biomarker and ***X***_*i-*_ includes the remaining ones in the set. Now the goal is to test the following hypothesis:

*H*_0*c*_: *Given* ***X***_*i-*_*, X*_*i*_*has no* discriminatory power to the disease.

The coefficients of the optimal linear combination of ***X*** are written as $a^{\;*T}=\left(a_{i-}^{*T},a_{i}^{*}\right)$, where $a_{i}^{*}$ is the corresponding coefficient of *X*_*i*_. In this problem, we propose evaluating the biomarker *X*_*i*_ from $a_{i}^{*}$. Given ***X***_*i-*_*,* this biomarker has no discriminatory power to the disease, if it does not contribute to the linear combination in terms of having a zero coefficient. That is, *H*_0*,c*_ is equivalent to

$$H_{0,c}:a_{i}^{*}=0$$

The test statistic is the estimator of $a_{i}^{*},$ denoted by $T_{c,i}={\hat{a}}_{n,i}$. The null hypothesis *H*_0*,c*_ is then rejected if *T*_*c,* *i*_ is either too small or too large.

To generate the bootstrap samples, the null scenario under *H*_0*,c*_ is discussed. Under the normality assumption, given *D* = *d*, *d* ∈ {0, 1},

$$X=\left(\begin{array}{c} X_{i-}\\ X_{i} \end{array}\right)|D=d\sim \mathrm{MVN}\left(\left(\begin{array}{c} \mu_{d,i-}\\ \mu_{d,i} \end{array}\right),\left(\begin{array}{cc} \Sigma_{d,i-} & \Sigma_{d,i-i}\\ \Sigma_{d,i-i}^{T} & \Sigma_{d,i} \end{array}\right)\right).$$

Then in *H*_0*,c*_*P*(*X*_*i*_|*D*, ***X***_*i* -_) = *P*(*X*_*i*_| ***X***_*i* -_), which holds providing that for each realization, ***X***_*i-*_ = ***x***_*i-*_*,*

$$\begin{array}{c} \mu_{1,i}+\Sigma_{1,i-i}^{T}\Sigma_{1,i-}^{-1}\left(x_{i-}-\mu_{1,i-}\right)=\mu_{0,i}+\Sigma_{0,i-i}^{T}\Sigma_{0,i-}^{-1}\left(x_{i-}-\mu_{0,i-}\right),\\ \sigma_{1,i}-\Sigma_{1,i-i}^{T}\Sigma_{1,i-}^{-1}\Sigma_{1,i-i}=\sigma_{0,i}-\Sigma_{0,i-i}^{T}\Sigma_{0,i-}^{-1}\Sigma_{0,i-i} \end{array}$$

Therefore, estimating the null distribution involves a non-trivial constrained inference. For simplicity, we consider a narrower null scenario, where *P*(*X*_*i*_|*D*, ***X***_*i* -_) = *P*(*X*_*i*_). That is, within the two groups, not only does *X*_*i*_ have a common distribution, but *X*_i_ is also independent from ***X***_*i-*_*.* As a consequence, we then consider the following model for bootstrap samples: for *d* = 0,1,

$$\left(X\right|D=d∼\mathrm{MVN}\left(\left(\begin{array}{c} {\hat{\mu }}_{d,i-}\\ {\tilde{\mu }}_{p,i} \end{array}\right),\left(\begin{array}{c} {\hat{\Sigma }}_{d,i-}\;0\\ 0^{T}\;{\tilde{\sigma }}_{p,i} \end{array}\right)\right).$$

Notations ${\hat{\mu }}_{d,i-}$ and ${\hat{\Sigma }}_{d,i-}$ represent the MLEs of the mean and covariance matrix of ***X***_*i-*_ respectively from the two samples; ${\tilde{\mu }}_{p,i},{\tilde{\sigma }}_{p,i}$ are estimates of the mean and variance of *X*_*i*_ from the pooled sample; **0** is the (*p*-1) x 1 zero vector. Repeat the bootstrap sampling *B* times, find the sample pAUC maximizers of the bootstrap samples, and record the *B* estimated coefficient ${\hat{a}}_{n,i}^{\left(b\right)}$ correspondent to *X*_*i*_. The critical values are then the 100 (α/2)^*th*^ and the 100(1-α/2)^*th*^ percentiles among the *B* coefficients. The null hypothesis is rejected if the test statistic *T*_*c,i*_ is greater than or equal to the 100 (1-α/2)^*th*^ percentile, or is less than or equal to the 100 (α/2)^*th*^ percentile.

Note that this conditional test is powerless to detect the significance of *X*_*i*_ when ***X***_*i-*_ solely is independent of the disease *D*. Under *H*_0*,c*_, it’s known that

$$P\left(X_{i},X_{i-}|D\right)=P\left(X_{i}|\;X_{i-}\right)P\left(\;X_{i-}|D\right).$$

Combining the fact that *P*(***X***_*i-*_*|D*) *= P*(***X***_*i-*_), it then leads to the complete null scenario that all biomarkers are independent of the disease. Under the circumstance, the estimated coefficients have great variability subject to the requirement of unit length in the algorithm. As a consequence, the critical values become so extreme that obtaining a significant finding is unlikely, even when in fact *X*_*i*_ is strongly correlated with the disease.

### Biomarker selection

We now turn to the biomarker selection problem. By using the statistical tests in the last subsection, we are able to determine the significance of a biomarker. The amount of data is reduced by selecting the significant biomarkers.

Assume that ***X*** is the vector of the full biomarker set and let ${\hat{a}}_{n}^{T}=\left({\hat{a}}_{n,1},\ldots ,{\hat{a}}_{n,p}\right)$ be the estimate of the optimal linear combination as before. We then employ the idea of a classical stepwise variable selection method. First, an ordering criterion for all biomarkers is determined. Here, the biomarkers are rearranged according to their corresponding $\left|{\hat{a}}_{n,i}\right|$ values in ascending order. The ordered biomarker set is denoted by ***X***^*T*^ *= (X*_(1)_*,…, X*_(*p*)_*)*. Hence, *X*_(1)_ is potentially the least important biomarker and *X*_(*p*)_ is potentially the most important one. Note that the ordering criterion is reasonable only when all biomarkers are expressed in a common unit, hence an adequate standardization should be applied before we proceed to the selection procedure.

We consider two stepwise selection methods: the Forward and the Backward approaches. For convenience, define *A* as the set of biomarkers under consideration for the disease diagnosis in each step. The Forward procedure starts with a null *A*, and tests the contribution of the potentially most discriminatory biomarker *X*_(*p*)_. The biomarker is added to *A* if it is significant. Then it consecutively assesses *X*_(*p*-1)_, *X*_(*p*-2)_ and so on. On the other hand, the Backward procedure begins with testing the overall discriminatory power of *A* = {***X***}. If there is a significant global effect, one further determines whether the potentially least discriminatory biomarker *X*_(1)_ is significant. Remove the biomarker from *A* if an insignificant result is present. Given the result, this procedure consecutively assesses the conditional contribution of *X*_(2)_, of *X*_(3)_ and so on. The details are presented below:

Forward method

Step 1. Set *A* = Ø. Test the marginal effect of *X*_(*p*)_ with respect to

*H*_*0,*(*p*)_ : *X*_(*p*)_*has no discriminatory power.*

If *H*_0*,*(*p*)_ is rejected, add *X*_(*p*)_ to *A*.

Go to the next step.

Step 2. Test the significance of *X*_(*p*-1)_ with respect to

*H*_0(*p*-1):_*Given A, X*_(*p*-1)_*has no discriminatory power.*

If *H*_0*,*(*p*-1)_ is rejected, add *X*_(*p*-1)_ to *A*.

Go to the next step.

Step p. Test the significance of *X*_(1)_ with respect to

*H*_0*,*(1)_: *Given A, X*_(1)_*has no discriminatory power.*

If *H*_0,(1)_ is rejected, add *X*_(1)_ to *A.*

Stop.

Backward method

Step 0. Set *A* = {***X***}. Test the global effect of *A* with respect to

*H*_0,(0)_: *A has no discriminatory power.*

If *H*_0,(0)_ is rejected, go to the next step; otherwise, stop and conclude *A* = Ø.

Step 1. Assess *X*_(1)_ by removing *X*_(1)_ from *A* and test the hypothesis,

*H*_0,(1)_: *Given A, X*_(1)_*has no discriminatory power.*

If *H*_0,(1)_ is rejected, add *X*_(1)_ to *A*.

Go to the next step.

Step 2. Assess *X*_(2)_ by removing *X*_(2)_ from *A* and test the hypothesis,

*H*_0,(2)_: *Given A, X*_(2)_*has no discriminatory power.*

If *H*_0,(2)_ is rejected, add *X*_(2)_ to *A*.

Go to the next step.

⋮

Step p. Assess the effect of *X*_(*p*)_. If *A* = {*X*_(*p*)_}, stop; otherwise, remove *X*_(*p*)_ from *A* and test the following null hypothesis,

*H*_0,(*p*)_: *Given A, X*_(*p*)_*has no discriminatory power.*

If *H*_0,(*p*)_ is rejected, add *X*_(*p*)_ to *A.*

Stop.

In the end of the selection process, we conclude that the biomarkers in *A* have a significant contribution to disease diagnosis. At Step 0 of the Backward approach, the global test is conducted; see *H*_0,*g*_ and *T*_*g*_ in Section 3.1. Moreover, during the selection, in testing the contribution of a specific biomarker, two different tests are applied depending on whether *A* is empty or not. If *A* = Ø, this is the problem of testing the marginal contribution of the target biomarker; see *H*_0*,m*_ and *T*_*m,i*_ in Section 3.1. If *A* ≠ Ø, then the conditional contribution of the target biomarker is tested; see *H*_0,*c*_ and *T*_*c,i*_ in Section 3.1.

For a study of *p* biomarkers, the Forward approach needs *p* tests for the final conclusion. However, the Backward approach is not that simple. It might stop immediately at Step 0 if an insignificant global discriminatory power is obtained. When the global significance is achieved and the first *p* - 1 biomarkers have all been concluded to be insignificant, we directly draw the conclusion of selecting only *X*_(*p*)_ without verifying its significance. If none of the above is the case, the evaluation of *X*_(*p*)_ is necessary. Hence, the Backward approach may take 1, *p* or *p* + 1 test(s) to reach its final conclusion. The stepwise method, which combines the forward and the backward selections, is another potential approach. However, it will take much longer computational time.

Sometimes a biomarker has no discriminatory power by itself, but has a contribution given the existence of other biomarkers. The contribution mainly comes from high correlations with other major biomarkers. In a selection procedure, this biomarker is likely to be selected. However, given this biomarker, the conditional test is powerless to detect other important biomarkers, as described in the last subsection. As a consequence, the Backward approach may produce a confusing conclusion: select a minor biomarker but discard a major one. On the other hand, because the Forward approach starts by assessing the marginal contribution of every biomarker, it tends to yield less positive findings if the effect sizes or the pAUCs of the biomarkers are small to moderate. In the next section, we will further explain these findings by way of a simulation study and real examples.

## Results

In this section, we perform simulation results to validate our proposed procedures, including the estimation of the best linear combination of the biomarkers, the global test of the discriminatory power of a set of biomarkers, and the two biomarker selection approaches. We generate samples of two, three and four biomarkers (*p* = 2,3,4) in various scenarios. To prevent the report from becoming too lengthy, we only provide a discussion on the case of two biomarkers and partial results for the cases of three and four biomarkers. More numerical results are provided in the additional files (see Additional file 1).

In the following, given the parameters values, the true best linear combinations maximizing the pAUC are found via grid-search with 10^6^ grids. When the data dimension *p ≤* 2, fixed grids are considered. When the data dimension is greater than two, the grids are drawn uniformly on the surface of a sphere [22,23]. On the other hand, based on the sample data, the estimated best linear combinations are computed via the multiple-initial algorithm proposed in our previous study [13].

Assume that the two biomarkers ***X*** = (*X*_1_,*X*_2_)^T^, given *D = d*, follow a bivariate-normal distribution with mean ***μ***_*d*_ and covariance **Σ**_*d*_, where *d =* 0 or 1 indicates a non-diseased or diseased group, respectively. Suppose that ***μ***_0_ = **0** and consequently, **μ**_1_ is equal to the mean difference, ***μ***_1_ = **Δ** = (Δ_1_,Δ_2_)^*T*^. Three values, 0.3, 0.5, and 1 are considered for Δ_*i*_’s. To mimic a standardized data set, the two biomarkers have unit variance, and correlation coefficient *ρ*_*d*_*.* The correlation coefficient *ρ*_*d*_ takes on one of three values: 0, 0.5 or 0.9, see Table 1. Consider the pAUC with *t* = 0.1. Table 1 also reports the distribution of a^*T^ ***X*** in the two groups. Further, the last column displays the true maximal pAUC values attained.

**Table 1:** The setting of populations

	**Mean difference**		**Correlation**		**Coefficients**		**Non-diseased**		**Diseased**		
**Case**	**∆**_**1**_	**∆**_**2**_	***ρ***_**0**_	***ρ***_**1**_	a^*^		a^*T^ ***μ***_**0**_	***Q***_**0**_	a^*T^ ***μ***_**1**_	***Q***_**1**_	***pAUC*** (a^*^)
1	0.0	0.0	0.0	0.0	0.00	0.00	NA	NA	NA	NA	0.0050
2	0.0	0.3	0.0	0.0	0.00	1.00	0.00	1.00	0.30	1.00	0.0088
3	0.0	0.5	0.0	0.0	0.00	1.00	0.00	1.00	0.50	1.00	0.0123
4	0.0	1.0	0.0	0.0	0.00	1.00	0.00	1.00	1.00	1.00	0.0245
5	0.0	0.3	0.5	0.5	-0.45	0.89	0.00	0.60	0.27	0.60	0.0095
6	0.0	0.5	0.5	0.5	-0.45	0.89	0.00	0.60	0.45	0.60	0.0138
7	0.0	1.0	0.5	0.5	-0.45	0.89	0.00	0.60	0.89	0.60	0.0292
8	0.0	0.3	0.9	0.9	-0.67	0.74	0.00	0.10	0.22	0.10	0.0163
9	0.0	0.5	0.9	0.9	-0.67	0.74	0.00	0.10	0.37	0.10	0.0290
10	0.0	1.0	0.9	0.9	-0.67	0.74	0.00	0.10	0.74	0.10	0.0690
11	0.0	0.3	0.5	0.0	-0.65	0.77	0.00	0.51	0.23	1.00	0.0164
12	0.0	0.5	0.5	0.0	-0.61	0.80	0.00	0.52	0.40	1.00	0.0204
13	0.0	1.0	0.5	0.0	-0.52	0.86	0.00	0.56	0.86	1.00	0.0333
14	0.0	0.3	0.9	0.0	-0.69	0.72	0.00	0.10	0.22	1.00	0.0367
15	0.0	0.5	0.9	0.0	-0.68	0.73	0.00	0.10	0.37	1.00	0.0422
16	0.0	1.0	0.9	0.0	-0.66	0.76	0.00	0.11	0.75	1.00	0.0567
17	0.0	0.3	0.0	0.5	0.56	0.83	0.00	1.00	0.25	1.46	0.0119
18	0.0	0.5	0.0	0.5	0.47	0.88	0.00	1.00	0.44	1.41	0.0148
19	0.0	1.0	0.0	0.5	0.24	0.98	0.00	1.00	0.97	1.23	0.0256
20	0.0	0.3	0.0	0.9	0.60	0.80	0.00	1.00	0.24	1.87	0.0144
21	0.0	0.5	0.0	0.9	0.53	0.85	0.00	1.00	0.42	1.81	0.0172
22	0.0	1.0	0.0	0.9	0.33	0.95	0.00	1.00	0.95	1.55	0.0270
23	0.3	0.3	0.0	0.0	0.71	0.71	0.00	1.00	0.43	1.00	0.0109
24	0.5	0.5	0.0	0.0	0.71	0.71	0.00	1.00	0.71	1.00	0.0167
25	1.0	1.0	0.0	0.0	0.71	0.71	0.00	1.00	1.41	1.00	0.0380

The first case is the complete null scenario, where the two biomarkers have the same distribution in the diseased and non-diseased groups. Each linear combination provides no discriminatory power to the disease and has the reference pAUC value *t*^2^ /2 = 0.005. Define a^*^ = **0** in this case. In Case 2–22, Δ_1_ = 0, Δ_2_ > 0, hence the second biomarker is the dominant biomarker. In Case 2–4, the two biomarkers are conditionally independent, and thus the first biomarker is completely uncorrelated with the disease while the second biomarker is the only contributor to the disease diagnosis. In Case 5–10, we find that the first biomarker can provide a non-ignorable contribution when it is correlated with the major contributor. Comparing this with Case 2–4, we observe that the global discriminatory power is significantly increased by the presence of the positive correlation. To further investigate the effect of correlation, we consider various covariance matrices. The two biomarkers are correlated only in the non-diseased group in Case 11–16, and only in the diseased group in Case 17–22. It can be seen that the existence of a positive correlation in the non-diseased group has a greater improvement in pAUC than in the diseased group. In the last three cases, Δ_1_ = Δ_2_, *ρ*_*d*_ = 0, and hence both biomarkers are of equal importance. The pAUC of the best linear combination increases with the common mean difference as expected.

Next, we study the empirical performances of the proposed estimated best linear combination $\left({\hat{a}}_{n}\right)$ and the correspondent pAUC $\left(\mathrm{pAUC}\left({\hat{a}}_{n}\right)\right)$. Consider a balanced study, in which *n*_0_ = *n*_1_ = 100. In Table 2, the empirical mean and standard error of these estimators among 1,000 replicates, denoted by Ave and SE, are reported.

**Table 2:** The related optimal coefficients a^*^, *pAUC* (a^*^), and the power of the global test

	$a_{1}^{*}$			$a_{2}^{*}$			**pAUC**			
**Case**	**True**	**Ave**	**SE**	**True**	**Ave**	**SE**	**True**	**Ave**	**SE**	**Power(*T***_**g**_**)**
1	0.000	-0.014	0.707	0.000	0.046	0.706	0.005	0.008	0.002	0.043
2	0.000	-0.005	0.552	1.000	0.763	0.337	0.009	0.011	0.003	0.271
3	0.000	0.016	0.427	1.000	0.892	0.147	0.012	0.014	0.004	0.631
4	0.000	0.018	0.238	1.000	0.970	0.042	0.025	0.026	0.006	0.999
5	-0.447	-0.331	0.473	0.894	0.779	0.245	0.010	0.011	0.003	0.349
6	-0.447	-0.400	0.294	0.894	0.859	0.126	0.014	0.015	0.004	0.731
7	-0.447	-0.428	0.129	0.894	0.892	0.059	0.029	0.030	0.006	1.000
8	-0.669	-0.655	0.098	0.743	0.746	0.067	0.016	0.018	0.004	0.895
9	-0.669	-0.666	0.039	0.743	0.744	0.035	0.029	0.030	0.006	0.999
10	-0.669	-0.668	0.019	0.743	0.743	0.017	0.069	0.070	0.006	1.000
11	-0.645	-0.569	0.324	0.765	0.694	0.299	0.016	0.017	0.004	0.907
12	-0.606	-0.598	0.116	0.795	0.787	0.100	0.020	0.021	0.004	0.995
13	-0.519	-0.514	0.088	0.855	0.852	0.052	0.033	0.034	0.005	1.000
14	-0.692	-0.659	0.215	0.722	0.689	0.212	0.037	0.037	0.004	1.000
15	-0.682	-0.680	0.050	0.731	0.730	0.049	0.042	0.043	0.004	1.000
16	-0.657	-0.656	0.024	0.754	0.754	0.020	0.057	0.057	0.005	1.000
17	0.563	0.407	0.441	0.826	0.686	0.412	0.012	0.013	0.003	0.505
18	0.467	0.436	0.238	0.884	0.853	0.157	0.015	0.016	0.004	0.799
19	0.239	0.234	0.163	0.971	0.958	0.042	0.026	0.027	0.005	0.999
20	0.604	0.451	0.438	0.797	0.653	0.423	0.014	0.015	0.004	0.792
21	0.529	0.498	0.232	0.848	0.812	0.195	0.017	0.018	0.004	0.923
22	0.325	0.326	0.123	0.946	0.936	0.044	0.027	0.028	0.005	0.999
23	0.707	0.603	0.364	0.707	0.607	0.368	0.011	0.013	0.003	0.478
24	0.707	0.664	0.241	0.707	0.667	0.237	0.017	0.018	0.004	0.903
25	0.707	0.696	0.117	0.707	0.698	0.117	0.038	0.039	0.007	1.000

In estimating the best linear combination, we find that it tends to give conservative results that are biased towards zero. The estimators have the greatest variations in the complete null scenario, and the variations decrease as the discriminating power of the two biomarkers increases. The estimated pAUC tends to overestimate the true value, and similarly this tendency increases as the set of the two biomarkers have a greater diagnostic power. As suggested by a referee, the use of an independent validation test set can be expected to reduce the over-estimation. The last column displays the empirical power of the global discriminatory power test at significance level α = 5% with bootstrapping size 500. We find that the test controls the type I error rate well and has satisfactory performance in alternative cases.

Next, we apply the two biomarker selection approaches. At each step, the significance level is α = 5% and the bootstrapping size is 500. There are four possible conclusions: (i) (*c*_*1*_*,c*_*2*_), if both biomarkers are selected; (ii) (1,0), if only the first biomarker is selected; (iii) (0,1), if only the second is selected; (iv) (0,0), if both are discarded. If at least one biomarker is selected, the best linear combination of the reduced biomarker set, as well as its correspondent pAUC value, is solved. The mean and the standard error of the maximal pAUC among the non-empty reduced sets are reported in Table 3. Table 4 lists the proportions of the four possible conclusions of the two approaches among the 1,000 replications. In each scenario, the figure in boldface corresponds to the most likely outcome.

**Table 3:** The pAUC and pAUC estimate after the biomarker-selection

		**Forward selection**		**Backward selection**	
**Case**	***pAUC*** (a^*^)	**Ave**	**SE**	**Ave**	**SE**
1	0.0050	0.0106	0.0016	0.0114	0.0018
2	0.0088	0.0120	0.0023	0.0129	0.0025
3	0.0123	0.0137	0.0032	0.0150	0.0033
4	0.0245	0.0250	0.0054	0.0248	0.0056
5	0.0095	0.0119	0.0024	0.0122	0.0030
6	0.0138	0.0140	0.0034	0.0138	0.0036
7	0.0292	0.0276	0.0080	0.0180	0.0099
8	0.0163	0.0125	0.0039	0.0092	0.0031
9	0.0290	0.0172	0.0093	0.0100	0.0040
10	0.0690	0.0628	0.0192	0.0077	0.0101
11	0.0164	0.0119	0.0026	0.0095	0.0027
12	0.0204	0.0145	0.0049	0.0118	0.0038
13	0.0333	0.0305	0.0091	0.0123	0.0095
14	0.0367	0.0149	0.0096	0.0085	0.0032
15	0.0422	0.0203	0.0141	0.0099	0.0048
16	0.0567	0.0526	0.0139	0.0075	0.0082
17	0.0119	0.0122	0.0028	0.0114	0.0027
18	0.0148	0.0135	0.0032	0.0139	0.0036
19	0.0256	0.0251	0.0056	0.0248	0.0059
20	0.0144	0.0120	0.0028	0.0102	0.0027
21	0.0172	0.014	0.0039	0.0128	0.0038
22	0.0270	0.0251	0.0059	0.0236	0.0067
23	0.0109	0.0123	0.0025	0.0131	0.0025
24	0.0167	0.0159	0.0047	0.0157	0.0044
25	0.0380	0.0387	0.0071	0.0387	0.0070

**Table 4:** The proportion of outcomes from the two biomarker selection methods among 1000 replications

			**Forward method**				**Backward method**			
**Case**	$a_{1}^{*}$	$a_{2}^{*}$	**(*c***_**1,**_ ***c***_**2**_**)**	**(1,0)**	**(0,1)**	**(0,0)**	**(*c***_**1,**_ ***c***_**2**_**)**	**(1,0)**	**(1,0)**	**(0,0)**
1	0.000	0.000	0.001	0.036	0.051	**0.912**	0.000	0.019	0.024	**0.957**
2	0.000	1.000	0.002	0.040	0.416	**0.542**	0.001	0.042	0.228	**0.729**
3	0.000	1.000	0.005	0.012	**0.799**	0.184	0.003	0.031	**0.597**	0.369
4	0.000	1.000	0.015	0.000	**0.984**	0.001	0.007	0.006	**0.986**	0.001
5	-0.447	0.894	0.008	0.021	0.424	**0.547**	0.000	0.064	0.285	**0.651**
6	-0.447	0.894	0.011	0.007	**0.788**	0.194	0.002	0.066	**0.663**	0.269
7	-0.447	0.894	0.355	0.000	**0.645**	0.000	0.038	0.330	**0.632**	0.000
8	-0.669	0.743	0.030	0.000	0.394	**0.576**	0.006	0.242	**0.647**	0.105
9	-0.669	0.743	0.189	0.001	**0.613**	0.195	0.011	0.340	**0.648**	0.001
10	-0.669	0.743	**0.884**	0.000	0.115	0.001	0.026	**0.891**	0.083	0.000
11	-0.645	0.765	0.008	0.027	0.412	**0.553**	0.001	0.231	**0.675**	0.093
12	-0.606	0.795	0.077	0.008	**0.713**	0.202	0.009	0.167	**0.819**	0.005
13	-0.519	0.855	**0.622**	0.000	0.377	0.001	0.037	**0.614**	0.349	0.000
14	-0.692	0.722	0.062	0.013	0.380	**0.545**	0.006	0.300	**0.694**	0.000
15	-0.682	0.731	0.200	0.001	**0.593**	0.206	0.012	0.337	**0.651**	0.000
16	-0.657	0.754	**0.898**	0.000	0.102	0.000	0.027	**0.876**	0.097	0.000
17	0.563	0.826	0.013	0.030	0.430	**0.527**	0.001	0.074	0.430	**0.495**
18	0.467	0.884	0.015	0.019	**0.769**	0.197	0.006	0.057	**0.736**	0.201
19	0.239	0.971	0.027	0.000	**0.973**	0.000	0.015	0.020	**0.964**	0.001
20	0.604	0.797	0.011	0.023	0.417	**0.549**	0.004	0.149	**0.639**	0.208
21	0.529	0.848	0.034	0.006	**0.775**	0.185	0.012	0.086	**0.825**	0.077
22	0.324	0.946	0.073	0.000	**0.926**	0.001	0.025	0.059	**0.915**	0.001
23	0.707	0.707	0.011	0.304	**0.367**	0.318	0.005	0.234	0.239	**0.522**
24	0.707	0.707	0.165	0.391	**0.408**	0.036	0.113	**0.402**	0.388	0.097
25	0.707	0.707	**0.965**	0.014	0.021	0.000	**0.964**	0.017	0.019	0.000

From Table 3, we can see that the Forward approach generally outperforms the Backward approach except in the null case. When the first biomarker has a non-ignorable contribution mainly due to the existence of a positive correlation between the two biomarkers, such as in Case 7–16, the Backward approach has unsatisfactory performance. From Table 4, we find that in these cases, a quite certain proportion of samples select only the first biomarker, which in fact has no marginal discriminatory power at all. More specifically, after obtaining a significant global effect at step 0, the potentially less important biomarker, which is likely the first one in the simulation, is assessed. We often obtain significance due to the obvious decrease in pAUC caused by removing the biomarker. Next, the conditional discriminatory power of the second biomarker, given the first biomarker, is assessed. As explained in Section 3, the conditional test is powerless when the given biomarker is independent of the disease. Thus, this major biomarker is likely discarded after the minor biomarker is selected.

On the other hand, in these scenarios the Forward approach, which begins by assessing the most discriminatory biomarker, is not able to derive the benefits from the correlation, and has less positive discoveries, as seen in Case 8–9, 11–12 and 14–15. However, as the effect size of the biomarker increases, the Forward approach has adequate power in identification of both important biomarkers, and hence it has better performance in terms of achievement of pAUC as seen in Table 3.

To investigate the robustness of our methods with respect to deviation from the binormality assumption, we generate 1,000 random samples of two biomarkers from multivariate-t distributions with degree of freedom 3. In Table 5, the true maximal pAUC value, *pAUC*(a^*^), is found via a grid search under the multivariate-t distribution. Additionally, we report the average and the standard error of the estimated maximal pAUC value of the reduced biomarker set, which is selected via our proposed methods on the basis of binormality. We find that in this case, our methods tend to produce optimistic conclusions. The proposed pAUC estimation and the resultant biomarker selection procedures are sensitive to the binormality assumption.

**Table 5:** The related pAUCs based on multivariate t distribution with degree of freedom 3

**Population (*X*)**					**pAUC**			
**Mean difference**		**Correlation**			**Forward selection**		**Backward selection**	
**∆**_**1**_	**∆**_**2**_	***ρ***_**0**_	***ρ***_**1**_	pAUC(a^*^ ***)***	**Ave**	**SE**	**Ave**	**SE**
0.0	0.3	0.5	0.5	0.0070	0.0159	0.0057	0.0143	0.0062
0.0	0.5	0.5	0.5	0.0088	0.0165	0.0058	0.0140	0.0063
0.0	1.0	0.5	0.5	0.0160	0.0205	0.0074	0.0166	0.0083
0.0	0.3	0.5	0.0	0.0116	0.0175	0.0069	0.0108	0.0066
0.0	0.5	0.5	0.0	0.0136	0.0187	0.0079	0.0121	0.0075
0.0	1.0	0.5	0.0	0.0206	0.0224	0.0089	0.0147	0.0093
0.0	0.3	0.0	0.5	0.0088	0.0166	0.0058	0.0137	0.0062
0.0	0.5	0.0	0.5	0.0101	0.0168	0.0060	0.0143	0.0066
0.0	1.0	0.0	0.5	0.0150	0.0197	0.0067	0.0178	0.0073
0.3	0.3	0.0	0.0	0.0076	0.0158	0.0051	0.0149	0.0055
0.5	0.5	0.0	0.0	0.0101	0.0169	0.0059	0.0158	0.0063
1.0	1.0	0.0	0.0	0.0206	0.0241	0.0086	0.0225	0.0094

Next, we study the cases consisting of three and four biomarkers (*p* = 3 or 4). Again, assume ***μ***_0_ = **0** and ***μ***_1_ = **Δ** = (Δ_1_,…,Δ_*p*_)^*T*^. Further, the covariance matrices are of the following form: for *d* = 0,1,

$$\begin{array}{ll} \mathrm{if}\;p & =3,\Sigma_{d}=\left(\begin{array}{ccc} 1 & \rho_{d} & 0\\ \rho_{d} & 1 & 0\\ 0 & 0 & 1 \end{array}\right),\mathrm{and}\;\mathrm{if}\;p=4,\Sigma_{d}\\ & =\left(\begin{array}{cccc} 1 & \rho_{d} & 0 & 0\\ \rho_{d} & 1 & 0 & 0\\ 0 & 0 & 1 & 0\\ 0 & 0 & 0 & 1 \end{array}\right). \end{array}$$

The performance of the estimated pAUC of the best linear combination of the full biomarker set, and that of the reduced biomarker set found from the two biomarker selection approaches, are presented in Table 6. Similar to the cases of *p* = 2, we can see that the estimated pAUC tends to overestimate the true value. By using the Backward approach, we are less likely to obtain a confusing conclusion as in the case of *p* = 2. Currently, the two selection approaches have comparable performance in most cases, except Case 11 of *p* = 3 and Case 8 of *p* = 4.

**Table 6:** The related pAUCs and the global test for three and four dimensions

								**Full biomarker Set**				**Reduced biomarker Set**			
		**Mean difference**				**Correlation**						**Forward selection**		**Backward selection**	
	**Case**	**∆**_**1**_	**∆**_**2**_	**∆**_**3**_	**∆**_**4**_	***ρ***_**0**_	***ρ***_**1**_	**True**	**Ave**	**SE**	**Power (*T***_**g**_**)**	**Ave**	**SE**	**Ave**	**SE**
*p =* 3	1	0.0	0.0	0.0	-	0.0	0.0	0.005	0.010	0.002	0.052	0.011	0.002	0.013	0.002
	2	0.5	0.0	0.0	-	0.0	0.0	0.012	0.015	0.004	0.576	0.014	0.003	0.015	0.003
	3	0.5	0.5	0.0	-	0.0	0.0	0.017	0.019	0.004	0.870	0.016	0.005	0.016	0.005
	4	0.5	0.5	0.5	-	0.0	0.0	0.021	0.023	0.005	0.977	0.018	0.006	0.018	0.007
	5	0.5	1.0	0.0	-	0.0	0.0	0.028	0.030	0.006	1.000	0.028	0.007	0.027	0.008
	6	0.5	0.5	0.0	-	0.1	0.1	0.016	0.018	0.004	0.845	0.015	0.004	0.016	0.004
	7	0.5	0.5	0.0	-	0.5	0.5	0.014	0.016	0.004	0.713	0.015	0.004	0.016	0.004
	8	0.5	0.5	0.0	-	0.9	0.9	0.013	0.015	0.004	0.616	0.014	0.004	0.016	0.004
	9	0.5	1.0	0.0	-	0.1	0.1	0.027	0.029	0.006	0.998	0.027	0.006	0.027	0.007
	10	0.5	1.0	0.0	-	0.5	0.5	0.025	0.026	0.006	0.995	0.025	0.006	0.025	0.006
	11	0.5	1.0	0.0	-	0.9	0.9	0.036	0.038	0.006	1.000	0.034	0.010	0.021	0.017
*p =* 4	1	0.0	0.0	0.0	0.0	0.0	0.0	0.005	0.011	0.002	0.051	0.011	0.002	0.012	0.002
	2	0.5	0.0	0.0	0.0	0.0	0.0	0.012	0.016	0.004	0.520	0.014	0.004	0.015	0.003
	3	0.5	0.5	0.0	0.0	0.0	0.0	0.017	0.020	0.004	0.862	0.016	0.005	0.016	0.005
	4	0.5	1.0	0.0	0.0	0.0	0.0	0.028	0.031	0.006	1.000	0.028	0.007	0.026	0.008
	5	0.5	1.0	1.0	0.0	0.0	0.0	0.041	0.043	0.007	1.000	0.042	0.008	0.042	0.008
	6	0.5	1.0	0.0	0.0	0.1	0.1	0.027	0.029	0.006	0.995	0.027	0.007	0.026	0.007
	7	0.5	1.0	0.0	0.0	0.5	0.5	0.025	0.027	0.005	0.993	0.025	0.006	0.025	0.006
	8	0.5	1.0	0.0	0.0	0.9	0.9	0.036	0.038	0.006	1.000	0.035	0.009	0.021	0.017
	9	0.5	1.0	1.0	0.0	0.1	0.1	0.040	0.042	0.007	1.000	0.041	0.008	0.041	0.008
	10	0.5	1.0	1.0	0.0	0.5	0.5	0.038	0.040	0.006	1.000	0.039	0.007	0.039	0.008
	11	0.5	1.0	1.0	0.0	0.9	0.9	0.048	0.050	0.007	1.000	0.047	0.009	0.049	0.008

### Applications to real data sets

We apply our procedures to some real examples in [10,24,25]. The 1-specificity upper limit is t = 0.1, the stepwise significance level is α = 5%, and the bootstrapping size is 500 during the biomarker selection. We use a multiple-initial algorithm to find the estimated best linear combinations of these real examples [13]. Before the biomarker selection, standardization is conducted. After subtracting the non-diseased group mean, every biomarker is divided by its pooled sample standard deviation from the two groups for a more constant unit across biomarkers. In addition, the analytical results of the data without standardization can be found in the additional files (see Additional file 1). With regard to the distributional assumption, it has been concluded that the first two example data sets do not deviate significantly from the binormality in their original papers [10,24]. However, in the last example, we obtain significant evidence (p-value < 0.0000) against the normality hypothesis for both samples via the package myShapiroTest of R software. Although the binormality assumption fails, this data set is still analyzed to demonstrate the applicability of our proposed methods to larger data sets. The famous algorithm-based variable selection method, LASSO, is also applied to this example for comparison.

The first example is a study of Duchenne Muscular Dystrophy (DMD) [24]. The DMD carriers generally are elevated by certain serum enzymes, not by physical symptoms. The measurements of 3 biomarkers of DMD of 87 normal and 38 carrier females were collected in this data set. The sample means of the three biomarkers in the normal and carrier groups are, respectively,

$$\begin{array}{l} {\hat{\mu }}_{0}={\left(3.393,4.521,2.486\right)}^{T},\\ {\hat{\mu }}_{1}={\left(4.762,4.523,3.011\right)}^{T}; \end{array}$$

and the sample covariance matrices are

$$\begin{array}{l} {\hat{\Sigma }}_{0}=\left(\begin{array}{ccc} 0.032 & -0.004 & 0.002\\ -0.004 & 0.007 & 0.001\\ 0.002 & 0.001 & 0.011 \end{array}\right),\\ {\hat{\Sigma }}_{1}=\left(\begin{array}{ccc} 0.768 & -0.005 & 0.305\\ -0.005 & 0.009 & -0.006\\ 0.305 & -0.006 & 0.227 \end{array}\right). \end{array}$$

Table 7 presents the results of biomarker selection. Both the Forward and Backward approaches select the first and the third biomarkers. We find that the decrease in the pAUC, which occurs when removing the second biomarker, is slim. The stepwise details are provided in Table 8.

**Table 7:** The estimated best linear combination and the corresponding pAUC in DMD and heart disease examples

**Case**	**Method**	${\hat{a}}_{1}$	${\hat{a}}_{2}$	${\hat{a}}_{3}$	${\hat{a}}_{4}$	${\hat{\mathrm{pAUC}}}_{n}$
DMD	Full set (raw)	0.8350	0.5116	0.2026	-	0.0888
	Full set (Standardized)	0.9895	0.0653	0.1292	-	0.0888
	Forward Selection	0.9657	0.0000	0.2597	-	0.0885
	Backward Selection	0.9657	0.0000	0.2597	-	0.0885
Heart disease	Full set (raw)	0.9447	0.3258	0.0265	0.0274	0.0165
	Full set (Standardized)	0.7079	0.6754	0.0834	0.1890	0.0165
	Forward Selection	1.0000	0.0000	0.0000	0.0000	0.0099
	Backward Selection	1.0000	0.0000	0.0000	0.0000	0.0099

**Table 8:** The Forward and Backward selections in DMD and heart disease examples

I. Forward selection						
Example	Step	Marker enters	Test statistic	Test value	p-value	Marker selected
DMD	1	*X*_*1*_	$\hat{\mathrm{pAUC}}$	0.0882	0.000^*^	*X*_*1*_
	2	*X*_*3*_	${\hat{a}}_{3}$	0.1775	0.006^*^	*X*_*1*_*,X*_*3*_
	3	*X*_*2*_	${\hat{a}}_{2}$	0.0653	0.272	*X*_*1*_*,X*_*3*_
Heart Disease	1	lutein	$\hat{\mathrm{pAUC}}$	0.0099	0.012^*^	lutein
	2	TBARS	${\hat{a}}_{T\mathrm{BARS}}$	0.7922	0.082	lutein
	3	uric acid	${\hat{a}}_{\mathrm{uric}\;\mathrm{acid}}$	0.5091	0.258	lutein
	4	HDL Chol	${\hat{a}}_{\mathrm{HDL}\;C}$	0.3352	0.428	lutein
II. Backward selection						
Example	Step	Marker enters	Test statistic	Test value	p-value	Marker selected
DMD	1	All	$\hat{\mathrm{pAUC}}$	0.0888	0.000^*^	*X*_*1*_*,X*_*2*_*,X*_*3*_
	2	*X*_*2*_	${\hat{a}}_{2}$	0.0653	0.272	*X*_*1*_*,X*_*3*_
	3	*X*_*3*_	${\hat{a}}_{3}$	0.1775	0.006^*^	*X*_*1*_*,X*_*3*_
	4	*X*_*1*_	${\hat{a}}_{1}$	0.9841	0.000^*^	*X*_*1*_*,X*_*3*_
Heart Disease	1	All	$\hat{\mathrm{pAUC}}$	0.0165	0.002^*^	lutein,TBARS, HDL Chol, uric acid
	2	HDL Chol	${\hat{a}}_{\mathrm{HDL}\;C}$	0.0834	0.632	lutein, TBARS, uric acid
	3	uric acid	${\hat{a}}_{\mathrm{uricacid}}$	0.1916	0.316	lutein, TBARS
	4	TBARS	${\hat{a}}_{T\mathrm{BARS}}$	0.7922	0.100	lutein

Note: * indicates a significance of α = 5%.

Another real example, four biomarkers (lutein, TBARS, HDL cholesterol, and uric acid) are used for construction of a classification tool for atherosclerotic coronary heart disease [10]. A cohort of 434 subjects, which includes 72 cases and 362 controls, was selected for the analysis. One obtains an insignificant conclusion in testing the null hypothesis of normality. For the non-diseased and diseased groups, the estimated means of the four markers are

$$\begin{array}{l} {\hat{\mu }}_{0}={\left(0.128,0.885,4.077,6.772\right)}^{T},\\ {\hat{\mu }}_{1}={\left(0.140,0.934,4.123,6.911\right)}^{T} \end{array}$$

and the two sample covariance matrices are

$$\begin{array}{c} {\hat{\Sigma }}_{0}=\left(\begin{array}{cccc} 0.003 & -0.000 & -0.000 & -0.005\\ -0.000 & 0.029 & 0.004 & 0.042\\ -0.000 & 0.004 & 0.049 & 0.027\\ -0.005 & 0.042 & 0.027 & 0.285 \end{array}\right)\\ {\hat{\Sigma }}_{1}=\left(\begin{array}{cccc} 0.004 & 0.003 & 0.007 & 0.007\\ 0.003 & 0.042 & 0.002 & 0.043\\ 0.007 & 0.002 & 0.039 & 0.001\\ 0.007 & 0.043 & 0.001 & 0.150 \end{array}\right). \end{array},$$

From Table 7, we obtain a different optimal linear combination of the full data set, in which the impact of the first biomarker lutein is diminished, while those of the other three are increased. Before the biomarker selection, the first two biomarkers, lutein and TBARS, seem to be important to the disease as evidenced by the magnitudes of their coefficients. However, after the biomarker selection, the two stepwise selections produce the same conclusion that only the biomarker lutein achieves statistical significance, as seen in Table 7 and 8.

The third example consists of 106 breast tissue samples [25]. Among them, 54 are classified as diseased and 52 as non-diseased. Nine biomarkers are available. The data can be downloadable from the additional files (see Additional file 2, [26]). Table 9 reports the results of the two biomarker selections of the standardized data. The biomarker set selected by the Forward method surpasses the set selected by the Backward method. Further, the two methods select two different sets of significant biomarkers. While the Backward approach discards the biomarkers more likely to be in the bottom group (in terms of the magnitude of the correspondent coefficient in the optimal linear combination of the full data set), the Forward approach does not select the four biomarkers with the largest coefficients in the full model. The latter implies an inconsistency between the coefficient of the optimal linear combination and the marginal discriminatory power of a biomarker. From an in-depth investigation, we found that in these top four biomarkers the non-diseased population is far more varied than the diseased population (see Additional file 1). This leads to a low pAUC value and hence an insignificance in testing the marginal discriminatory power. In contrast, a biomarker with a more homogeneous non-diseased population is preferred under the pAUC criterion. Since our proposed methods do not terminate after an insignificant finding, the impact of the variable ordering during selection is narrowed.

**Table 9:** The estimated best linear combination and the corresponding pAUC in the breast tissue example

**Method**	**I0**	**PA500**	**HFS**	**DA**	**AREA**	**A/DA**	**MAX IP**	**DR**	**P**	${\hat{\mathrm{pAUC}}}_{n}$
Full set	-0.572	0.284	0.028	-0.296	-0.164	0.091	-0.038	0.391	0.560	0.059
Forward	0.000	0.821	0.000	0.000	-0.358	0.384	-0.223	0.000	0.000	0.058
Backward	-0.731	0.000	0.000	-0.109	-0.088	0.060	0.000	0.262	0.612	0.047
LASSO (λ_min_)	-0.572	0.284	0.028	-0.296	-0.164	0.091	-0.038	0.391	0.560	0.059
LASSO (λ_1SE_)	-0.088	0.992	0.000	0.000	0.000	-0.095	0.000	0.000	0.000	0.051

For a comparison, we also report the result of the optimal linear combination of the reduced biomarker sets, which are selected using the LASSO. Two different λ’s are used: the one achieving the minimum mean cross-validation error, denoted as λ_min_; and the maximal value such that the corresponding mean error is within 1 standard error of the minimum, denoted as λ_1SE_. From Table 9, we find that using λ_min_ in the LASSO produces the most conservative selection, in which none of the biomarkers are discarded. Using λ_1SE_, the LASSO selects a quite different biomarker set from those selected by our two approaches. This method is better than the Backward method but is surpassed by the Forward method for this application in terms of the sample maximal pAUC of the selected biomarker set. The analyses were performed by using the package cv.glment of R software with deviance loss and 10-fold cross-validation.

These three biomarkers of the third example, I0, A/DA and MAX IP were considered as the most discriminatory biomarkers in original paper [25]. From Table 9, we can observe that none of the biomarker sets selected by the discussed methods include all three biomarkers at the same time. One major reason for this is that the response, which originally had a more detailed categorization of six classes, is condensed into a binary variable here. Further, the objective function of original paper was the accuracy, while we consider the pAUC in this study [25]. Thus, different relevant statistical information is captured.

## Discussion

In this study, we focus on disease diagnosis with the presence of multiple biomarkers. We consider the class of linear combinations for an effective and easy-to-interpret summarization of the multiple biomarkers. The diagnostic power of a linear combination is evaluated based upon its pAUC over a clinically relevant threshold region. To be more precise, we consider the requirement of a high specificity for the purpose of population screening.

Under the binormality assumption, the pAUC of a linear combination is estimated via the employment of MLEs of the population parameters. In addition, the strong consistency of the estimated optimal linear combination is proved. We also introduce a testing procedure to assess the overall diagnostic power of a set of biomarkers based on the greatest pAUC it can achieve in the class of linear combinations. Furthermore, a testing procedure for determining the conditional contribution of a single biomarker given the existence of other biomarkers is developed. The parametric bootstrap method is applied to find the critical value(s) of the tests. These proposed tests are then embedded in two biomarker selection approaches. The finite sample performance of the proposed methods is studied by using both synthetic and real data sets. In addition, the robustness of our approaches with regard to the deviation from the binormality assumption is investigated via a simulation, and a comparison of our biomarker selection methods with the LASSO is conducted in a real data analysis.

Our methods differ with other algorithm-based marker-selection approaches in that we propose to select or discard a biomarker based upon evidence of statistical significance. As a trade-off, our methods involve many computations in order to acquire statistical evidence. This decreases the feasibility of applying these methods to larger data sets. Consequently, our methods are less appropriate in an exploratory study. We suggest the application of adequate data filtering for dimension reduction prior to advanced statistical confirmatory analysis, such as the construction of a diagnostic rule.

One common issue of selecting biomarkers based on the observed data is over-fitting. To prevent such a problem, one may use the method of cross-validation. This method can be easily applied to our proposed procedure. Hence, if the prediction power is the primary goal and the over-fitting is a concern in a real application, then the investigators can easily integrate the cross-validation method into our procedure. Although in this paper, we did not discuss more on over-fitting, the bootstrap resampling method we used in our procedure, which takes the sampling variation into account, can guard against over-fitting to some extents.

This research is conducted under the assumption that the biomarkers follow a multivariate normal distribution. The proposed statistical procedures are shown to be moderately sensitive to the distributional assumption via a numerical study. By using a non-parametric estimation of the pAUC as an alternative (for example, the empirical pAUC), the proposed methods can be generalized. But, theoretical verifications are still necessary for the resultant estimation of the optimizer. The non-smoothed functional form greatly increases computational difficulty. Development of non-parametric approaches may be more challenging, yet they can be more broadly applied. However, this topic is beyond the scope of our study.

Conventionally, a biomarker is often characterized by its mean and variance. However, from the simulation, we find that the correlation between biomarkers can play a critical role yet is often less emphasized. The pAUC of the linear combination of a set of biomarkers may be increased by including another biomarker, which is individually independent of the disease but highly correlated with other important biomarkers. The improvement of the pAUC can be substantial. Further, we observe that the correlation between biomarkers in the non-diseased group has a greater effect than that of in the diseased group. On the other hand, from the real example we observe that a biomarker with a more homogeneous non-diseased population is more likely to have a greater pAUC.

Before proceeding to the proposed test-based biomarker selection, suitable data standardization is recommended in order to have a fair ordering of the biomarkers by their coefficients in the best linear combination. Different standardizations can lead to different results in the best linear combination and hence differences in the ordering. However, in our methods, because all biomarkers enter the evaluation process and are assessed by incorporating their sampling variations, the effect of standardization is minimized. In fact, in the first two real examples of this study, the same conclusions are obtained with or without the standardization, which shows that our test-based procedures are robust with respect to the choice of standardization. The analysis of the raw data is provided in the additional files (see Additional file 1).

There are other options for ranking the biomarkers. For example, consider a ranking based on the association between every individual biomarker and the disease response measured by the p-value of a uni-variate t-test under the normality assumption. Or, because our article emphasizes the pAUC criterion, another possible ranking can be based upon the estimated marginal pAUC, as well as the sampling error, of a biomarker. However, these methods are more computationally intensive, and furthermore, they are unable to recognize associations between a biomarker and the disease in the presence of other biomarkers. Here, we propose using the coefficients of the optimal linear combination of the complete biomarker set as a ranking criterion. Our ranking criterion is relatively simple and roughly maps out biomarkers based on their importance. The limitation of this method is that in order to avoid the computational difficulty, the sampling error is not taken into consideration. We learn from one of the examples that an inconsistency between the coefficient of the optimal linear combination and the marginal discriminatory power may occur. Despite this, there is no criterion of an early stop and every biomarker is evaluated throughout the biomarker selection procedure in order to minimize the ranking effect.

As in a conventional regression analysis, we do not apply any multiplicity adjustment to strictly control a familywise type I error rate in the selection procedures. However, if the investigators require a more confirmatory conclusion, a multiplicity adjustment may be necessary. The Forward selection has a fixed number of steps, and hence it involves a simple multiple comparison problem. The conventional Bonferroni’s adjustment, by using the significance level *α/ p* at each step, can be applied directly. The Backward selection may take 1, *p* or *p* + 1 step(s) to reach the final conclusion. Then, the simplest and most conservative way is to use the significance level *α/* (*p* + 1) at each step for a control of the familywise error rate. Of course, with multiplicity adjustment, the comparison of the two biomarker selection approaches may yield different results.

## Conclusions

Our proposed biomarker selection approaches can be used to find the significant biomarkers based on hypothesis testing.

## Abbreviations

aSAH: Aneurysmal subarachnoid hemorrhage; PSA: Prostate-specific antigen; DMD: Duchenne muscular dystrophy.

## Competing interests

The authors declare that they have no competing interests.

## Authors’ contributions

All authors participated in the design and interpretation of the study. MH proved Theorem 1 and performed the simulation study. All authors contributed to the draft and have approved the final manuscript.

## Supplementary Material

Additional file 1 The proof of Theorem 1 and more numerical results. Click here for file

Additional file 2 Dataset with electrical impedance measurements in samples of freshly excised tissue from the breast. Click here for file
