# Supplementary material for: Biomarker selection for medical diagnosis using the partial area under the ROC curve
Source: BMC Res Notes. 2014 Jan 10;7:25. doi: 10.1186/1756-0500-7-25 (PMC3923449; doi:10.1186/1756-0500-7-25)
Supplement: Additional file 1 — The proof of Theorem 1 and more numerical results. [file 1756-0500-7-25-S1.pdf]

Biomarker Selection for Medical Diagnosis using the Partial Area under the ROC Curve

MAN-JEN HSU[1]*, YUAN-CHIN IVAN CHANG[1,2] and HUEY-MIIN HSUEH[2*]

In this supplement, we present the proof of Theorem 1 in Section 2, and more numerical results, including the simulation of three and four biomarkers ($p = 3{,}4$). On the other hand, the analysis results of the three real examples are provided.

1 The proof of Theorem 1

Theorem 1: Suppose that the conditional distribution of $\boldsymbol{X}|D = d$ follows $N(\boldsymbol{\mu}_d, \boldsymbol{\Sigma}_d)$ and $\boldsymbol{\Sigma}_d$ is positive definite for $d = 0{,}1$. Assume that $pAUC(\boldsymbol{a})$ in Equation (1) is a continuous function of $\boldsymbol{a}$ and has a unique maximizer $\boldsymbol{a}^*$ in E_p, and $\widehat{\boldsymbol{a}}_n$ is the maximizer of the sample pAUC, $\widehat{pAUC}_n(\boldsymbol{a})$, in Equation (2). Then $\widehat{\boldsymbol{a}}_n \to \boldsymbol{a}^*$ with probability 1 as $n \to \infty$.

Proof of Theorem 1.

Since E_p is compact, for any $\delta > 0$, there is a finite set $\{\boldsymbol{a}_1, \boldsymbol{a}_2, \ldots, \boldsymbol{a}_J\}$, with each $\boldsymbol{a}_j \in E_p$ such that $E_p \subseteq \bigcup_{j=1}^{J} \aleph(\boldsymbol{a}_j, \delta)$, where $\aleph(\boldsymbol{a}_j, \delta)$ is an open ball centered at $\boldsymbol{a}_j$ with radius δ. Then

$$\sup_{\boldsymbol{a}\in E_p} \left|\widehat{pAUC}_n(\boldsymbol{a}) - pAUC(\boldsymbol{a})\right|$$

$$= \max_j \sup_{\boldsymbol{a}\in E_p \cap \aleph(\boldsymbol{a}_j,\delta)} \left|\widehat{pAUC}_n(\boldsymbol{a}) - pAUC(\boldsymbol{a})\right|$$

$$\le \max_j \sup_{\boldsymbol{a}\in E_p \cap \aleph(\boldsymbol{a}_j,\delta)} \left|\widehat{pAUC}_n(\boldsymbol{a}) - \widehat{pAUC}_n(\boldsymbol{a}_j)\right|$$

$$+ \max_j \left|\widehat{pAUC}_n(\boldsymbol{a}_j) - pAUC(\boldsymbol{a}_j)\right|$$

$$+ \max_j \sup_{\boldsymbol{a}\in E_p \cap \aleph(\boldsymbol{a}_j,\delta)} \left|pAUC(\boldsymbol{a}) - pAUC(\boldsymbol{a}_j)\right|$$

$$\equiv (I) + (II) + (III) \qquad (S1)$$

By the mean value theorem and Cauchy-Schwarz inequality, for $\boldsymbol{a} \in E_p \cap \aleph(\boldsymbol{a}_j, \delta)$, there exists $\boldsymbol{c}_j$ between $\boldsymbol{a}$ and $\boldsymbol{a}_j$ such that

---

***Corresponding author:** 95354503@nccu.edu.tw, hsueh@nccu.edu.tw

[1] Institute of Statistical Science, Academia Sinica, Taipei 11529, Taiwan

[2] Department of Statistics, National ChengChi University, Taipei 11605, Taiwan

$$\left|\widehat{pAUC}_n(\boldsymbol{a}) - \widehat{pAUC}_n(\boldsymbol{a_j})\right| \le \left\|\frac{\partial \widehat{pAUC}_n(\boldsymbol{c_j})}{\partial \boldsymbol{a}}\right\| \cdot \delta \quad a.s..$$

Consequently,

$$(I) \le \sup_{\boldsymbol{a}\in E_p} \left\|\frac{\partial \widehat{pAUC}_n(\boldsymbol{a})}{\partial \boldsymbol{a}}\right\| \cdot \delta. \qquad (S2)$$

Lemma 1 below shows that $\widehat{pAUC}_n(\boldsymbol{a}) \to pAUC(\boldsymbol{a})$ with probability 1 for any fixed $\boldsymbol{a} \in E_p$. Hence, for each $j = 1, \dots, J$, there exists ϵ_j such that $n \to \infty$,

$$\left|\widehat{pAUC}_n(\boldsymbol{a_j}) - pAUC(\boldsymbol{a_j})\right| < \epsilon_j.$$

Let $\epsilon = \max_j \epsilon_j$, we have for sufficiently large n,

$$(II) < \epsilon. \qquad (S3)$$

Moreover, because $pAUC(\boldsymbol{a})$ is continuous at $\boldsymbol{a}$, there exists $\epsilon_j^* > 0$ such that, for any $\boldsymbol{a} \in E_p \cap \aleph(\boldsymbol{a}_j, \delta)$, $\left|pAUC(\boldsymbol{a}) - pAUC(\boldsymbol{a_j})\right| < \epsilon_j^*$. Take $\epsilon^* = \max_j \epsilon_j^*$. It implies that

$$(III) < \epsilon^*. \qquad (S4)$$

It follows from (S1) to (S4) that for any $\delta^* > 0$,

$$P\left(\text{limsup}_n \left\{\sup_{\boldsymbol{a}\in E_p} \left|\widehat{pAUC}_n(\boldsymbol{a}) - pAUC(\boldsymbol{a})\right| > \delta^*\right\}\right)$$

$$\le P\left(\text{limsup}_n \left\{ \sup_{\boldsymbol{a}\in E_p} \left\|\frac{\partial \widehat{pAUC}_n(\boldsymbol{a})}{\partial \boldsymbol{a}}\right\| \cdot \delta > \frac{\delta^*}{3}\right\}\right) + P\left(\epsilon > \frac{\delta^*}{3}\right) + P\left(\epsilon^* > \frac{\delta^*}{3}\right).$$

The last two terms vanish as long as ϵ, ϵ^* are chosen to satisfy $\epsilon < \frac{\delta^*}{3}, \epsilon^* < \frac{\delta^*}{3}$. Accordingly, the radius δ of the subcovers in the beginning is determined with such choices of ϵ, ϵ^*. Furthermore, by choosing $M_n = \frac{\delta^*}{3\delta}$ in Lemma 2 below, we find that the first term is negligible. Subsequently,

$$P\left(\limsup_n \left\{\sup_{\boldsymbol{a}\in E_p} \left|\widehat{pAUC}_n(\boldsymbol{a}) - pAUC(\boldsymbol{a})\right| > \delta^*\right\}\right) = 0. \qquad (S5)$$

Hence, $\widehat{pAUC}_n(\boldsymbol{a})$ converges to $pAUC(\boldsymbol{a})$ uniformly on E_p with probability 1. Moreover, since

$$\widehat{\boldsymbol{a}}_n = \arg\max_{\boldsymbol{a}\in E_p} \widehat{pAUC}_n(\boldsymbol{a}),\ \boldsymbol{a}^* = \arg\max_{\boldsymbol{a}\in E_p} pAUC(\boldsymbol{a}),$$

we have

$$\widehat{pAUC}_n(\widehat{\boldsymbol{a}}_n) \ge \widehat{pAUC}_n(\boldsymbol{a}^*), pAUC(\boldsymbol{a}^*) \ge pAUC(\widehat{\boldsymbol{a}}_n),$$

and

$$\begin{aligned} \widehat{pAUC}_n(\boldsymbol{a}^*) - pAUC(\boldsymbol{a}^*) &\le \widehat{pAUC}_n(\widehat{\boldsymbol{a}}_n) - pAUC(\boldsymbol{a}^*) \\ &\le \widehat{pAUC}_n(\widehat{\boldsymbol{a}}_n) - pAUC(\widehat{\boldsymbol{a}}_n). \end{aligned}$$

Then, by (S5)

$$\begin{aligned} &\left|\widehat{pAUC}_n(\widehat{\boldsymbol{a}}_n) - pAUC(\boldsymbol{a}^*)\right| \\ &\le \max\left\{\left|\widehat{pAUC}_n(\boldsymbol{a}^*) - pAUC(\boldsymbol{a}^*)\right|, \left|\widehat{pAUC}_n(\widehat{\boldsymbol{a}}_n) - pAUC(\widehat{\boldsymbol{a}}_n)\right|\right\} \\ &\le \sup_{\boldsymbol{a}\in E_p} \left|\widehat{pAUC}_n(\boldsymbol{a}) - pAUC(\boldsymbol{a})\right| \xrightarrow{a.s.} 0. \qquad (S6) \end{aligned}$$

Define Ω as the sample space and $\Omega_0 = \bigcap_{n=1}^{\infty} \bigcup_{m=n}^{\infty} \{\omega \in \Omega : \|\widehat{\boldsymbol{a}}_m(\omega) - \boldsymbol{a}^*\| > \epsilon\}$. Thus, if $\widehat{\boldsymbol{a}}_n$ fails to converge to $\boldsymbol{a}^*$ almost surely, then $P(\Omega_0) > 0$. Now, suppose that $P(\Omega_0) > 0$, then for all n, there is a $m_1 \geq n$ such that

$$\widehat{\boldsymbol{a}}_{m_1} = \arg\max_{\boldsymbol{a} \in E_p} \widehat{pAUC}_{m_1}(\boldsymbol{a}) \text{ and } \left\|\widehat{\boldsymbol{a}}_{m_1}(\omega) - \boldsymbol{a}^*\right\| > \epsilon \text{ for some } \omega \in \Omega_0.$$

However, because $pAUC(\boldsymbol{a})$ has a unique maximum, it follows that

$$\left|pAUC\left(\widehat{\boldsymbol{a}}_{m_1}(\omega)\right) - pAUC(\boldsymbol{a}^*)\right| > \epsilon^* \text{ for some } \epsilon^* > 0.$$

On the other hand, by (S5), for sufficiently large m_1,

$$\left|\widehat{pAUC}_{m_1}(\widehat{\boldsymbol{a}}_{m_1}) - pAUC(\widehat{\boldsymbol{a}}_{m_1})\right| \xrightarrow{a.s.} 0.$$

Hence, for a given $\epsilon^{**} > 0$, there exists an m_1 such that

$$\begin{aligned}
&\left|\widehat{pAUC}_{m_1}(\widehat{\boldsymbol{a}}_{m_1}) - pAUC(\boldsymbol{a}^*)\right| \\
&= \left|\left[pAUC(\widehat{\boldsymbol{a}}_{m_1}) - pAUC(\boldsymbol{a}^*)\right] - \left[pAUC(\widehat{\boldsymbol{a}}_{m_1}) - \widehat{pAUC}_{m_1}(\widehat{\boldsymbol{a}}_{m_1})\right]\right| \\
&\geq \left|\left|pAUC(\widehat{\boldsymbol{a}}_{m_1}) - pAUC(\boldsymbol{a}^*)\right| - \left|\widehat{pAUC}_{m_1}(\widehat{\boldsymbol{a}}_{m_1}) - pAUC(\widehat{\boldsymbol{a}}_{m_1})\right|\right| \\
&> \epsilon^{**},
\end{aligned}$$

which contradicts (S6). Hence, it implies that $\widehat{\boldsymbol{a}}_n \xrightarrow{a.s.} \boldsymbol{a}^*$. ■

Lemma 1: Suppose that the conditions in Theorem 1 hold, then for any fixed $\boldsymbol{a} \in E_p$, $\widehat{pAUC}_n(\boldsymbol{a}) \to pAUC(\boldsymbol{a})$ with probability 1.

Proof of Lemma 1.

Since $E(\boldsymbol{X}|D)^2 < \infty$, by SLLN, as $n \to \infty$,

$$\widehat{\boldsymbol{\mu}}_0 \xrightarrow{a.s.} \boldsymbol{\mu}_0,\ \ \widehat{\boldsymbol{\mu}}_1 \xrightarrow{a.s.} \boldsymbol{\mu}_1,\ \ \widehat{\boldsymbol{\Sigma}}_0 \xrightarrow{a.s.} \boldsymbol{\Sigma}_0,\ \ \widehat{\boldsymbol{\Sigma}}_1 \xrightarrow{a.s.} \boldsymbol{\Sigma}_1.$$

Consequently, for any fixed $\boldsymbol{a} \in E_p$,

$$\boldsymbol{a}^T\widehat{\boldsymbol{\Delta}}_\mu \xrightarrow{a.s.} \boldsymbol{a}^T\,\boldsymbol{\Delta}_\mu, \widehat{Q}_0 \xrightarrow{a.s.} Q_0, \widehat{Q}_1 \xrightarrow{a.s.} Q_1,$$

and

$$\widehat{F}_n(\boldsymbol{a}, u) = \Phi\left(\frac{\boldsymbol{a}^T\widehat{\boldsymbol{\Delta}}_\mu - c(u)\sqrt{\widehat{Q}_0}}{\sqrt{\widehat{Q}_1}}\right) \xrightarrow{a.s.} \Phi\left(\frac{\boldsymbol{a}^T\boldsymbol{\Delta}_\mu - c(u)\sqrt{Q_0}}{\sqrt{Q_1}}\right) = F(\boldsymbol{a}, u),$$

since $\Phi(\cdot)$ is a continuous function ([1], Theorem 1.10 (i)). Further, since $\Phi(\cdot)$ is bounded, by the dominated convergence theorem,

$$\lim_{n\to\infty} \widehat{pAUC}_n(\boldsymbol{a}) = \lim_{n\to\infty} \int_0^{\mathrm{t}} \widehat{F}_n(\boldsymbol{a}, u)\, du$$

$$= \int_0^{t} \lim_{n\to\infty} \hat{F}_n(\boldsymbol{a}, u)\, du$$

$$= \int_0^{t} F(\boldsymbol{a}, u)\, du = pAUC(\boldsymbol{a}).$$

Hence, for any fixed $\boldsymbol{a} \in E_p$, $\widehat{pAUC}_n(\boldsymbol{a}) \to pAUC(\boldsymbol{a})$ with probability 1. ■

Lemma 2: Suppose that the conditions in Theorem 1 hold, then $\left\|\frac{\partial \widehat{pAUC}_n(\boldsymbol{a})}{\partial \boldsymbol{a}}\right\|$ is uniformly bounded in $\boldsymbol{a} \in E_p$ with probability 1. That is, for any $\boldsymbol{a} \in E_p$, there exists M_n, which is free of $\boldsymbol{a}$ and converges as n goes to infinity, such that

$$P\left(\limsup_n \left\{\sup_{\boldsymbol{a}\in E_p} \left\|\frac{\partial \widehat{pAUC}_n(\boldsymbol{a})}{\partial \boldsymbol{a}}\right\| \geq M_n\right\}\right) = 0.$$

Proof of Lemma 2.

Define $\widehat{\boldsymbol{A}} = \frac{\boldsymbol{a}^T\widehat{\boldsymbol{\Delta}}_\mu}{\hat{Q}_1}\widehat{\boldsymbol{\Sigma}}_1\boldsymbol{a} - \widehat{\boldsymbol{\Delta}}_\mu$, $\widehat{\boldsymbol{B}} = \frac{\widehat{\boldsymbol{\Sigma}}_0\boldsymbol{a}}{\sqrt{\hat{Q}_0}} - \frac{\sqrt{\hat{Q}_0}}{\hat{Q}_1}\widehat{\boldsymbol{\Sigma}}_1\boldsymbol{a}$, $\hat{v} = \frac{\boldsymbol{a}^T\widehat{\boldsymbol{\Delta}}_\mu\sqrt{\hat{Q}_0}}{\hat{Q}_0+\hat{Q}_1}$, $\hat{\sigma}^2 = \frac{\hat{Q}_1}{\hat{Q}_0+\hat{Q}_1}$, $\widehat{W} = exp\left\{-\frac{(\boldsymbol{a}^T\widehat{\boldsymbol{\Delta}}_\mu)^2}{2(\hat{Q}_0+\hat{Q}_1)}\right\}\frac{1}{\sqrt{2\pi(\hat{Q}_0+\hat{Q}_1)}}$, and $\phi(\cdot)$ is the density function of the standard normal distribution. Then for any fixed $\boldsymbol{a} \in E_p$, as $n \to \infty$,

$$\left\|\frac{\partial \widehat{pAUC}_n(\boldsymbol{a})}{\partial \boldsymbol{a}}\right\| = \left\|\frac{\partial}{\partial \boldsymbol{a}}\int_0^{t}\left[1-\Phi\left(\frac{c(u)\sqrt{\hat{Q}_0}-\boldsymbol{a}^T\widehat{\boldsymbol{\Delta}}_\mu}{\sqrt{\hat{Q}_1}}\right)\right]du\right\|$$

$$=\left\|\int_0^{t}\phi\left(\frac{c(u)\sqrt{\hat{Q}_0}-\boldsymbol{a}^T\widehat{\boldsymbol{\Delta}}_\mu}{\sqrt{\hat{Q}_1}}\right)\frac{1}{\sqrt{\hat{Q}_1}}\left(\widehat{\boldsymbol{A}}+\widehat{\boldsymbol{B}}c(u)\right)du\right\|$$

$$=\left\|\widehat{W}\left[(\widehat{\boldsymbol{A}}+\widehat{\boldsymbol{B}}\hat{v})\Phi\left(\frac{\hat{v}-c(t)}{\hat{\sigma}}\right)+\widehat{\boldsymbol{B}}\frac{1}{\sqrt{2\pi}}exp\left\{-\frac{(\hat{v}-c(t))^2}{2\hat{\sigma}^2}\right\}\right]\right\|$$

$$\equiv M^*(\boldsymbol{a}).$$

Since $\widehat{\boldsymbol{\Sigma}}_0$, $\widehat{\boldsymbol{\Sigma}}_1$ both are symmetric positive definite matrices, and $\|\boldsymbol{a}\| = 1$, then

$$0 < \lambda_p \leq \hat{Q}_0 \leq \lambda_1, 0 < \lambda_p^* \leq \hat{Q}_1 \leq \lambda_1^*,$$

where λ_p, λ_1, and λ_p^*, λ_1^* are the smallest and the largest eigenvalues of $\widehat{\boldsymbol{\Sigma}}_0$, $\widehat{\boldsymbol{\Sigma}}_1$, respectively ([2], Theorem 3.15). Consequently,

$$M^*(\boldsymbol{a}) \leq \left\|\widehat{W}\left(\widehat{\boldsymbol{A}}+\widehat{\boldsymbol{B}}\hat{v}\right)\right\| + \left\|\widehat{W}\widehat{\boldsymbol{B}}\right\|$$

$$\leq \frac{\boldsymbol{a}^T\widehat{\boldsymbol{\Delta}}_\mu\|\widehat{\boldsymbol{\Sigma}}_0+\widehat{\boldsymbol{\Sigma}}_1\|}{(\hat{Q}_0+\hat{Q}_1)^{3/2}} + \frac{\|\widehat{\boldsymbol{\Delta}}_\mu\|}{\sqrt{\hat{Q}_0+\hat{Q}_1}} + \frac{\hat{Q}_1\|\widehat{\boldsymbol{\Sigma}}_0\|+\hat{Q}_0\|\widehat{\boldsymbol{\Sigma}}_1\|}{\sqrt{\hat{Q}_0+\hat{Q}_1}\sqrt{\hat{Q}_0}\hat{Q}_1}$$

$$\leq \frac{\|\widehat{\boldsymbol{\Delta}}_\mu\|\|\widehat{\boldsymbol{\Sigma}}_0+\widehat{\boldsymbol{\Sigma}}_1\|}{\left(\lambda_p+\lambda_p^*\right)^{3/2}} + \frac{\|\widehat{\boldsymbol{\Delta}}_\mu\|}{\sqrt{\lambda_p+\lambda_p^*}} + \frac{\lambda_1^*\|\widehat{\boldsymbol{\Sigma}}_0\|+\lambda_1\|\widehat{\boldsymbol{\Sigma}}_1\|}{\sqrt{\lambda_p+\lambda_p^*}\sqrt{\lambda_p}\lambda_p^*}$$

$$\equiv M_n,$$

where M_n is free of $\boldsymbol{a}$ and converges as n goes to infinity. Hence, $\left\|\frac{\partial \widehat{pAUC}_n(\boldsymbol{a})}{\partial \boldsymbol{a}}\right\|$ is uniformly bounded in $\boldsymbol{a}$ with probability 1. ■

2. Numerical Study

2.1 Simulations of Three and Four Biomarkers

Consider $p = 3,4$. Assume $\boldsymbol{\mu}_0 = \mathbf{0}$ in the non-diseased group, and $\boldsymbol{\mu}_1 = \boldsymbol{\Delta} = (\Delta_1, \dots, \Delta_p)^T$ in the diseased group. Further, the covariance matrices are of the following form: for $d = 0, 1$,

$$if\ p = 3, \boldsymbol{\Sigma}_d = \begin{pmatrix} 1 & \rho_d & 0 \\ \rho_d & 1 & 0 \\ 0 & 0 & 1 \end{pmatrix}, and\ if\ p = 4, \boldsymbol{\Sigma}_d = \begin{pmatrix} 1 & \rho_d & 0 & 0 \\ \rho_d & 1 & 0 & 0 \\ 0 & 0 & 1 & 0 \\ 0 & 0 & 0 & 1 \end{pmatrix}.$$

The population setting can be found in Table 5 of the article. In Table S1, the true value of the best linear combination; empirical mean and standard error of the estimated $\widehat{\boldsymbol{a}}_n$ based on 1000 replications, denoted by True, AVE and SE, are reported. Table S2, S3 and S4 present the proportion of outcomes from the two biomarker selections among 1000 replicates. Table S2 reports the cases of $p = 3$, while Table S3 and S4 give the cases of $p = 4$. For three biomarkers, there are eight possible conclusions: (i) $(0,0,0)$, if all biomarkers are insignificant; (ii) $(1,0,0)$, if only the first biomarker is selected; (iii) $(0,1,0)$, if only the second is selected; (iv) $(0,0,1)$, if only the third is selected; (v) $(c_1, c_2, 0)$, if the first and the second are selected; (vi) $(c_1, 0, c_3)$, if the first and the third are selected; (vii) $(0, c_2, c_3)$, if the second and the third are selected; (viii) (c_1, c_2, c_3), if all biomarkers are selected. Table S2 lists the proportions of the eight possible conclusions of the two approaches among the 1000 replications for three dimension. For four biomarkers, there are sixteen possible conclusions: (i) $(0,0,0,0)$, if all biomarkers are insignificant; (ii) $(1,0,0,0)$, if only the first biomarker is selected; (iii) $(0,1,0,0)$, if only the second is selected; (iv) $(0,0,1,0)$, if only the third is selected; (v) $(0,0,0,1)$, if only the forth is selected; (vi) $(c_1, c_2, 0,0)$, if the first and the second are selected; (vii) $(c_1, 0, c_3, 0)$, if the first and the third are selected; (viii) $(c_1, 0,0, c_4)$, if the first and the forth are selected; (ix) $(0, c_2, c_3, 0)$, if the second and the third are selected; (x) $(0, c_2, 0, c_4)$, if the second and the forth are selected; (xi) $(0,0, c_3, c_4)$, if the third and the forth are selected; (xii) $(c_1, c_2, c_3, 0)$, if only the forth is not selected; (xiii) $(c_1, c_2, 0, c_4)$, if only the third is not selected; (xiv) $(c_1, 0, c_3, c_4)$, if only the second is not selected; (xv) $(0, c_2, c_3, c_4)$, if only the first is not selected; (xvi) (c_1, c_2, c_3, c_4), if all biomarkers are selected. The proportions of the sixteen possible conclusions of the two approaches among the 1000 replications are reported in Table S3 and Table S4, respectively. In each scenario, the figure in

boldface is correspondent to the most likely outcome.

2.2 Applications to Real Data Sets

Table S5 gives the two biomarker selection results of the DMD and atherosclerotic coronary heart disease examples by using the raw data. In the DMD example, $\boldsymbol{X_1}, \boldsymbol{X_3}$ are selected. In the heart disease example, only the lutein is concluded as a statistically significant biomarker.

The stepwise details of the standardized data of the breast tissue example are given in Table S6. In order to investigate the relationship between the pAUC and the marginal distributions of the individual biomarkers, we report the sample mean and variance within the two groups, as well as the pAUC in Table S7 in descending order based on the absolute value of the coefficient in the optimal linear combination of the full data set. Additionally, the corresponding density plots of each biomarker are given in Figure 1- Figure 9. In which the reference vertical line, x=c, is found from the given upper limit t=0.1 of the 1-specificity, and given the cutoff, the pAUC integrates all tailed probabilities in the diseased distribution. Moreover, Table S8 and Figures 10-11 present the characteristics of the optimal linear combinations of the reduced biomarkers set found by the two selection methods. Given the results, we found that a biomarker which has a homogeneous non-diseased population and a heterogeneous diseased population tends to have a higher pAUC value.

Table S1. The true value, empirical mean, and standard error of the estimated $\hat{\mathbf{a}}_{\mathrm{n}}$.

	Case	a_1^*			a_2^*			a_3^*			a_4^*		
		True	Ave	SE	True	Ave	SE	True	Ave	SE	True	Ave	SE
$p = 3$	1	0.000	0.015	0.578	0.000	0.003	0.577	0.000	0.008	0.578	-	-	-
	2	1.000	0.803	0.207	0.000	-0.002	0.378	0.000	-0.017	0.412	-	-	-
	3	0.707	0.613	0.241	0.707	0.637	0.233	0.000	0.023	0.324	-	-	-
	4	0.578	0.534	0.223	0.578	0.536	0.225	0.578	0.528	0.221	-	-	-
	5	0.440	0.420	0.181	0.898	0.861	0.097	0.000	-0.001	0.201	-	-	-
	6	0.706	0.620	0.254	0.708	0.614	0.262	0.000	0.007	0.325	-	-	-
	7	0.706	0.553	0.359	0.708	0.523	0.376	0.000	-0.011	0.389	-	-	-
	8	0.709	0.395	0.536	0.705	0.384	0.533	0.000	0.002	0.354	-	-	-
	9	0.371	0.367	0.202	0.928	0.876	0.103	0.000	-0.001	0.216	-	-	-
	10	0.000	0.032	0.256	1.000	0.937	0.077	0.000	0.009	0.221	-	-	-
	11	-0.584	-0.578	0.053	0.812	0.811	0.035	0.000	0.005	0.069	-	-	-
$p = 4$	1	0.000	0.022	0.494	0.000	0.026	0.499	0.000	0.025	0.514	0.000	-0.031	0.491
	2	1.000	0.751	0.205	0.000	0.003	0.359	0.000	-0.011	0.369	0.000	-0.014	0.358
	3	0.707	0.614	0.226	0.707	0.595	0.238	0.000	-0.006	0.290	0.000	0.000	0.279
	4	0.440	0.400	0.200	0.898	0.801	0.199	0.000	-0.001	0.196	0.000	-0.012	0.194
	5	0.330	0.333	0.138	0.657	0.642	0.117	0.678	0.639	0.114	0.000	0.004	0.154
	6	0.371	0.355	0.200	0.928	0.857	0.102	0.000	0.005	0.211	0.000	-0.001	0.212
	7	0.000	0.028	0.253	1.000	0.911	0.094	0.000	-0.009	0.220	0.000	-0.004	0.221
	8	-0.584	-0.579	0.053	0.812	0.807	0.034	0.000	0.002	0.073	0.000	-0.001	0.068
	9	0.274	0.277	0.154	0.668	0.639	0.117	0.692	0.663	0.112	0.000	0.003	0.159
	10	-0.001	0.021	0.186	0.717	0.672	0.125	0.697	0.675	0.117	0.000	0.000	0.170
	11	-0.554	-0.553	0.058	0.787	0.774	0.033	0.273	0.281	0.084	0.000	-0.003	0.067

Table S2. The proportion of outcomes from the two biomarker selection methods for three dimension.

Case	$(0,0,0)$	$(1,0,0)$	$(0,1,0)$	$(0,0,1)$	$(c_1,c_2,0)$	$(c_1,0,c_3)$	$(0,c_2,c_3)$	(c_1,c_2,c_3)
I. Forward selection								
1	**0.848**	0.057	0.046	0.047	0.000	0.001	0.001	0.000
2	0.194	**0.764**	0.013	0.023	0.003	0.003	0.000	0.000
3	0.045	0.400	**0.430**	0.009	0.105	0.002	0.002	0.007
4	0.006	0.228	**0.236**	0.224	0.075	0.065	0.057	0.109
5	0.000	0.022	**0.546**	0.000	0.405	0.000	0.012	0.015
6	0.037	**0.424**	0.410	0.017	0.101	0.003	0.002	0.006
7	0.075	**0.413**	0.396	0.021	0.087	0.006	0.001	0.001
8	0.149	0.367	**0.387**	0.010	0.083	0.003	0.000	0.001
9	0.000	0.011	**0.680**	0.000	0.292	0.000	0.010	0.007
10	0.001	0.002	**0.928**	0.001	0.053	0.000	0.015	0.000
11	0.000	0.000	0.193	0.000	**0.783**	0.000	0.001	0.023
II. Backward selection								
Case	$(0,0,0)$	$(1,0,0)$	$(0,1,0)$	$(0,0,1)$	$(c_1,c_2,0)$	$(c_1,0,c_3)$	$(0,c_2,c_3)$	(c_1,c_2,c_3)
1	**0.948**	0.014	0.020	0.018	0.000	0.000	0.000	0.000
2	0.424	**0.520**	0.026	0.025	0.004	0.000	0.000	0.001
3	0.130	0.364	**0.386**	0.021	0.083	0.002	0.002	0.012
4	0.023	0.219	**0.239**	0.220	0.050	0.054	0.047	0.148
5	0.000	0.056	**0.567**	0.003	0.344	0.000	0.008	0.022
6	0.155	0.356	**0.362**	0.024	0.095	0.002	0.001	0.005
7	0.287	**0.309**	0.298	0.028	0.064	0.003	0.005	0.006
8	**0.384**	0.278	0.272	0.021	0.037	0.001	0.003	0.004
9	0.002	0.031	**0.666**	0.009	0.273	0.000	0.005	0.014
10	0.005	0.007	**0.925**	0.010	0.044	0.000	0.006	0.003
11	0.000	0.373	0.194	0.000	**0.404**	0.007	0.000	0.022

Table S3. The proportion of outcomes from the Forward selection method among 1000 replications for four dimension.

Case	1	2	3	4	5	6	7	8	9	10	11
(0,0,0,0)	**0.808**	0.189	0.041	0.000	0.000	0.000	0.000	0.000	0.000	0.000	0.000
(1,0,0,0)	0.047	**0.727**	0.377	0.026	0.002	0.013	0.009	0.000	0.000	0.000	0.000
(0,1,0,0)	0.043	0.030	**0.412**	**0.582**	0.013	**0.665**	**0.904**	0.179	0.015	0.018	0.019
(0,0,1,0)	0.045	0.022	0.010	0.000	0.017	0.000	0.000	0.000	0.026	0.019	0.001
(0,0,0,1)	0.054	0.015	0.007	0.000	0.000	0.001	0.001	0.001	0.000	0.000	0.000
$(c_1, c_2, 0,0)$	0.001	0.005	0.134	0.344	0.004	0.274	0.045	**0.770**	0.003	0.000	0.006
$(c_1, 0, c_3, 0)$	0.000	0.006	0.002	0.000	0.001	0.000	0.000	0.000	0.005	0.006	0.000
$(c_1, 0,0, c_4)$	0.000	0.003	0.001	0.000	0.000	0.001	0.000	0.000	0.000	0.000	0.000
$(0, c_2, c_3, 0)$	0.001	0.000	0.008	0.012	0.404	0.014	0.014	0.000	**0.553**	**0.861**	0.141
$(0, c_2, 0, c_4)$	0.001	0.000	0.004	0.008	0.000	0.011	0.022	0.000	0.000	0.000	0.000
$(0,0, c_3, c_4)$	0.000	0.001	0.000	0.000	0.000	0.000	0.000	0.000	0.000	0.000	0.000
$(c_1, c_2, c_3, 0)$	0.000	0.000	0.001	0.017	**0.523**	0.007	0.002	0.025	0.369	0.067	**0.798**
$(c_1, c_2, 0, c_4)$	0.000	0.001	0.003	0.009	0.000	0.012	0.003	0.023	0.001	0.000	0.000
$(c_1, 0, c_3, c_4)$	0.000	0.001	0.000	0.000	0.000	0.000	0.000	0.000	0.000	0.000	0.000
$(0, c_2, c_3, c_4)$	0.000	0.000	0.000	0.001	0.014	0.002	0.000	0.000	0.015	0.026	0.005
(c_1, c_2, c_3, c_4)	0.000	0.000	0.000	0.001	0.022	0.000	0.000	0.002	0.013	0.003	0.030

Table S4. The proportion of outcomes from the Backward selection method among 1000 replications for four dimension.

Case	1	2	3	4	5	6	7	8	9	10	11
(0,0,0,0)	**0.949**	**0.480**	0.138	0.000	0.000	0.005	0.007	0.000	0.000	0.000	0.000
(1,0,0,0)	0.012	0.441	**0.382**	0.099	0.001	0.029	0.010	0.366	0.000	0.001	0.003
(0,1,0,0)	0.016	0.018	0.337	**0.520**	0.014	**0.661**	**0.896**	0.191	0.022	0.033	0.002
(0,0,1,0)	0.009	0.026	0.011	0.001	0.010	0.006	0.012	0.001	0.018	0.032	0.001
(0,0,0,1)	0.014	0.026	0.015	0.007	0.001	0.008	0.014	0.000	0.000	0.000	0.000
$(c_1, c_2, 0,0)$	0.000	0.001	0.086	0.318	0.001	0.257	0.040	**0.389**	0.002	0.000	0.002
$(c_1, 0, c_3, 0)$	0.000	0.003	0.001	0.000	0.003	0.000	0.000	0.003	0.006	0.002	0.003
$(c_1, 0,0, c_4)$	0.000	0.004	0.002	0.000	0.000	0.000	0.000	0.000	0.000	0.000	0.000
$(0, c_2, c_3, 0)$	0.000	0.000	0.002	0.004	0.389	0.003	0.002	0.000	**0.518**	**0.790**	0.001
$(0, c_2, 0, c_4)$	0.000	0.000	0.000	0.002	0.000	0.003	0.004	0.000	0.000	0.000	0.000
$(0,0, c_3, c_4)$	0.000	0.000	0.000	0.000	0.000	0.000	0.000	0.000	0.000	0.000	0.000
$(c_1, c_2, c_3, 0)$	0.000	0.000	0.015	0.020	**0.534**	0.011	0.009	0.031	0.392	0.085	**0.943**
$(c_1, c_2, 0, c_4)$	0.000	0.001	0.009	0.025	0.000	0.013	0.004	0.017	0.000	0.000	0.000
$(c_1, 0, c_3, c_4)$	0.000	0.000	0.002	0.000	0.000	0.000	0.000	0.000	0.000	0.002	0.000
$(0, c_2, c_3, c_4)$	0.000	0.000	0.000	0.001	0.016	0.002	0.002	0.000	0.030	0.049	0.000
(c_1, c_2, c_3, c_4)	0.000	0.000	0.000	0.003	0.031	0.002	0.000	0.002	0.012	0.006	0.045

Table S5. The Forward and Backward selections in DMD and atherosclerotic coronary heart disease examples.

I. Forward selection

Example	Step	Marker enters	Test statistic	Test value	p-value	Marker selected
DMD	1	X_1	$\widehat{pAUC}$	0.0882	0.000*	X_1
	2	X_2	$\hat{a}_2$	0.5236	0.212	X_1
	3	X_3	$\hat{a}_3$	0.2597	0.028*	X_1, X_3
Heart Disease	1	lutein	$\widehat{pAUC}$	0.0099	0.012*	lutein
	2	TBARS	$\hat{a}_{\mathrm{T}BARS}$	0.4036	0.076	lutein
	3	uric acid	$\hat{a}_{\text{uric acid}}$	0.0687	0.248	lutein
	4	HDL Chol	$\hat{a}_{\text{HDL C}}$	0.0999	0.410	lutein

II. Backward selection

Example	Step	Marker enters	Test statistic	Test value	p-value	Marker selected
DMD	1	All	$\widehat{pAUC}$	0.0888	0.000*	X_1, X_2, X_3
	2	X_3	$\hat{a}_3$	0.2026	0.026*	X_1, X_2, X_3
	3	X_2	$\hat{a}_2$	0.5116	0.192	X_1, X_3
	4	X_1	$\hat{a}_1$	0.9657	0.000*	X_1, X_3
Heart Disease	1	All	$\widehat{pAUC}$	0.0165	0.002*	lutein, TBARS, HDL Chol, uric acid
	2	HDL Chol	$\hat{a}_{\text{HDL C}}$	0.0265	0.690	lutein, TBARS, uric acid
	3	uric acid	$\hat{a}_{\text{uric acid}}$	0.0308	0.384	lutein, TBARS
	4	TBARS	$\hat{a}_{\mathrm{T}BARS}$	0.4036	0.098	lutein

Note: * indicates a significance at $\alpha = 5\%$.

Table S6. The Forward and Backward selections in breast tissue example.

I. Forward selection

Step	Marker enters	Test statistic	Test value	p-value	Marker selected
1	I0	$\widehat{pAUC}$	0.0002	1.000	
2	P	$\widehat{pAUC}$	0.0001	1.000	
3	DR	$\widehat{pAUC}$	0.0002	1.000	
4	DA	$\widehat{pAUC}$	0.0004	1.000	
5	PA500	$\widehat{pAUC}$	0.0490	0.000^{*}	PA500
6	AREA	$\hat{a}_{\mathrm{AREA}}$	-0.2470	0.038^{*}	PA500, AREA
7	A/DA	$\hat{a}_{\mathrm{A/DA}}$	0.2196	0.044^{*}	PA500, AREA, A/DA
8	MAX IP	$\hat{a}_{\mathrm{MAX\ IP}}$	-0.2234	0.026^{*}	PA500, AREA, A/DA, MAX IP
9	HFS	$\hat{a}_{\mathrm{HFS}}$	0.0873	0.372	PA500, AREA, A/DA, MAX IP

II. Backward selection

Step	Marker enters	Test statistic	Test value	p-value	Marker selected
1	All	$\widehat{pAUC}$	0.0591	0.000^{*}	I0, PA500, HFS, DA, AREA, A/DA, MAX IP, DR,P
2	HFS	$\hat{a}_{\mathrm{HFS}}$	0.0284	0.332	I0, PA500, DA, AREA, A/DA, MAX IP, DR, P
3	MAX IP	$\hat{a}_{\mathrm{MAX\ IP}}$	-0.0322	0.300	I0, PA500, DA, AREA, A/DA, DR, P
4	A/DA	$\hat{a}_{\mathrm{A/DA}}$	0.2859	0.004^{*}	I0, PA500, DA, AREA, A/DA, DR, P
5	AREA	$\hat{a}_{\mathrm{AREA}}$	-0.3082	0.010^{*}	I0, PA500, DA, AREA, A/DA, DR, P
6	PA500	$\hat{a}_{\mathrm{PA500}}$	0.5200	0.060	I0, DA, AREA, A/DA, DR, P
7	DA	$\hat{a}_{\mathrm{DA}}$	-0.1085	0.028^{*}	I0, DA, AREA, A/DA, DR, P
8	DR	$\hat{a}_{\mathrm{DR}}$	0.2616	0.012^{*}	I0, DA, AREA, A/DA, DR, P
9	P	$\hat{a}_{\mathrm{P}}$	0.6117	0.000^{*}	I0, DA, AREA, A/DA, DR, P
10	I0	$\hat{a}_{\mathrm{IO}}$	-0.7310	0.002^{*}	I0, DA, AREA, A/DA, DR, P

Note: * indicates a significance at $\alpha = 5\%$.

Table S7. The distributions of individual biomarkers for two groups; its marginal pAUC in breast tissue example.

	D=0		D=1		
Marker	mean	variance	mean	variance	$\widehat{pAUC}_n$
I0	0.0000	2.0024	-1.6187	0.0354	0.0002
P	0.0000	1.9912	-1.4715	0.0462	0.0001
DR	0.0000	1.8654	-0.8923	0.1673	0.0002
DA	0.0000	1.8721	-1.0244	0.1608	0.0004
PA500	0.0000	0.4252	1.1153	1.5531	0.0490
AREA	0.0000	2.0071	-0.5167	0.0309	0.0000
A/DA	0.0000	1.7155	-0.3562	0.3115	0.0001
MAX IP	0.0000	1.9371	-0.9185	0.0982	0.0000
HFS	0.0000	0.8450	0.2780	1.1491	0.0120

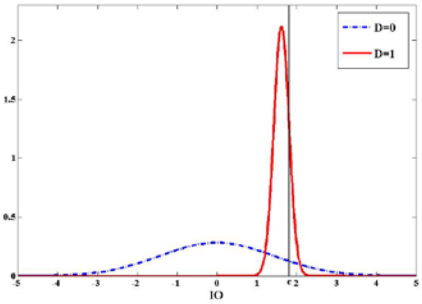

Figure 1. The distributions of I0 for two groups.

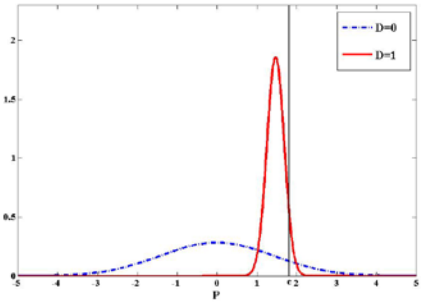

Figure 2. The distributions of P for two groups.

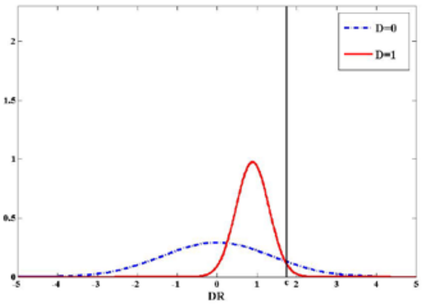

Figure 3. The distributions of DR for two groups.

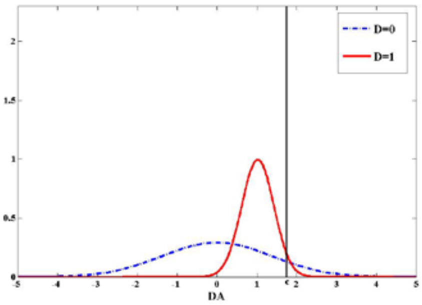

Figure 4. The distributions of DA for two groups.

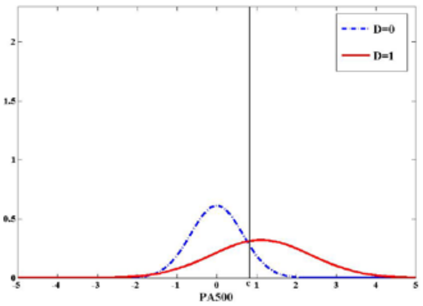

Figure 5. The distributions of PA500 for two groups.

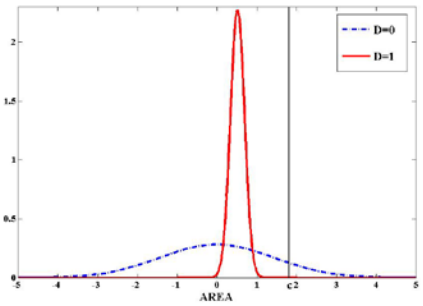

Figure 6. The distributions of AREA for two groups.

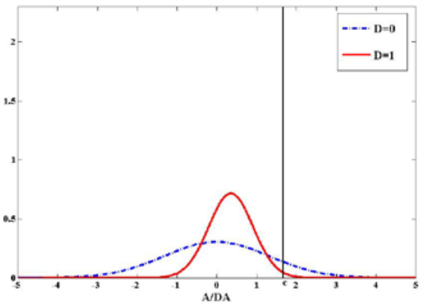

Figure 7. The distributions of A/DA for two groups.

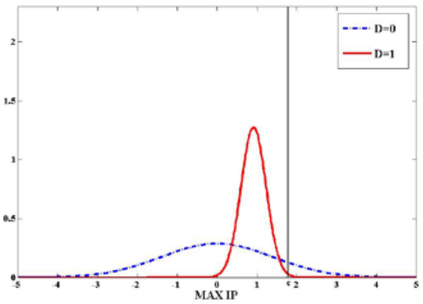

Figure 8. The distributions of MAX IP for two groups.

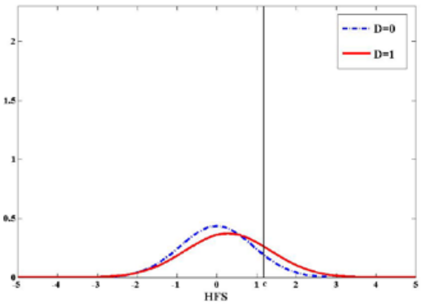

Figure 9. The distributions of HFS for two groups.

Table S8. The distributions of best linear combination for two groups; correspondent pAUC in breast tissue example.

	D=0		D=1		
Method	mean	variance	mean	variance	$\widehat{pAUC}_n$
Forward	0.0000	0.2949	1.1690	1.2116	0.0575
Backward	0.0000	0.0125	0.1852	0.0093	0.0466

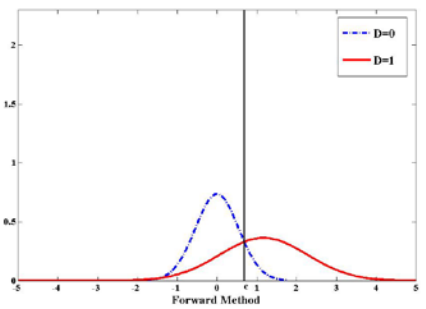

Figure 10. The distributions of best linear combination by the Forward method for two groups.

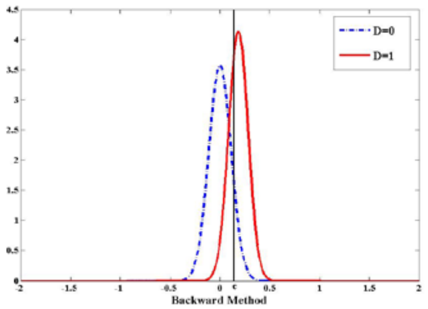

Figure 11. The distributions of best linear combination by the Backward method for two groups.

References

1. Schott, JR: Matrix Analysis For Statistics. Wiley Series in Probability and Statistics; 2005.

2. Shao, J: Mathematical Statistics. Springer-Verlag Inc; 1999.
